# FiberBrowser: a unified framework for single-molecule genomics

**DOI:** 10.64898/2026.10.29.762797

**Published:** 2026-10-31

**Authors:** Thomas W. Tullius, Christopher Catalano, Jane Ranchalis, Jie Hu, Mitchell R. Vollger, Andrew B. Stergachis, Michael S. Levine

**Affiliations:** 1 Lewis-Sigler Institute for Integrative Genomics, Princeton University, Princeton, NJ, USA.; 2 Department of Molecular Biology, Princeton University, Princeton, NJ, USA.; 3 Division of Medical Genetics, Department of Medicine, University of Washington, Seattle, WA, USA.; 4 Department of Human Genetics, University of Utah, Salt Lake City, UT, USA.; 5 Department of Genome Sciences, University of Washington, Seattle, WA, USA.; 6 Brotman Baty Institute for Precision Medicine, Seattle, WA, USA.

## Abstract

Single-molecule footprinting methods are rapidly transforming chromatin biology. They resolve nucleosome positioning, regulatory-factor occupancy, and accessibility on individual DNA fibers, revealing combinatorial heterogeneity that bulk measurements average away. Emerging footprinting methods leverage diverse enzyme systems and sequencing platforms, necessitating tools that can accommodate these systems. We present FiberBrowser, a unified platform that converts these diverse datasets into a common per-molecule representation of nucleosomes, accessible DNA, and subnucleosomal footprints for interactive and scripted analyses. Its inference engine, FiberHMM, weighs each observation against the assay’s informative sites and sequence-specific modification probabilities, separating missing information from protection in four assays. FiberBrowser clusters, sorts and compares these calls across thousands of molecules in seconds, linking chromatin architecture to haplotype, CpG methylation and non-reference structural variants on the same molecules. Because the representation is independent of chemistry, models also transfer between assays. A classifier trained on Fiber-seq footprints identifies 12 scDAF-seq cells as lymphoblastoid without retraining, whereas a model trained on raw modification patterns fails to transfer. FiberBrowser thus provides a unified, expandable analytical framework for single-molecule chromatin footprinting.

## Introduction

Single-molecule footprinting resolves nucleosome positioning, polymerase and transcription factor occupancy, and accessibility together on individual chromatin fibers, revealing combinatorial states that bulk short-read epigenomic methods average away^1,2^. Accessible DNA can be labeled by adenine methyltransferases (Fiber-seq, SAMOSA and others^3–6^), cytosine methyltransferases (SMF, NOMe-seq and others^7–12^) or cytosine deaminases (DAF-seq, FOODIE and others^13–16^), and the marks are read by short-read, PacBio or Oxford Nanopore (ONT) sequencing.

These assays read the same chromatin states, but their data differ. Each enzyme targets different bases with its own sequence preferences, and methyltransferase assays sequenced on PacBio without amplification report both strands, whereas ONT and amplified deaminase assays report only one^17,18^. Resolution therefore depends on how densely the target bases occur and on whether both strands are read. Detection also varies: ONT readouts of methyltransferase marks miss many modified bases, and mismatch-based readouts are prone to false-positive modification calls. These differences matter because a footprint is inferred from the absence of modification, so an unmodified stretch can mean either that a protein protected the DNA or that the assay had few chances to report a modification there. A caller that ignores this distinction reports false footprints where informative bases are sparse and misses short footprints that dense data support. Accurate footprinting therefore requires weighing every observation against where the assay could have reported a modification and how likely it was to do so.

Analysis tools have largely been developed alongside individual assays and for specific use cases. Fibertools (ft) calls m6A and manipulates, annotates and summarizes Fiber-seq data, and its simple heuristic-based nucleosome caller underlies Fiber-seq inference of regulatory elements (FIRE)^19,20^. IDLI classifies nucleosomal and subnucleosomal footprints in SAMOSA data^4,21^, and SingleMoleculeFootprinting and FootprintCharter call footprints from CpG and GpC methylation at selected loci^10,22–24^. Analyses accompanying FOODIE^14^, FootTrack for cFOOT-seq^16^ and HiddenFoot^25^ score protection at predefined intervals, in aggregate signal or at specified binding sites. Most of these tools are tied to one enzyme and platform, and several require motif or locus annotations in advance, which limits their use genome-wide and across diverse chromatin types. What remains missing is a factor-agnostic footprint caller that returns nucleosome and footprint intervals on individual molecules across chemistries, with an explicit model of what each assay can observe.

Visualization presents a second gap. General-purpose genome browsers such as IGV and the UCSC Genome Browser can display base modifications on individual long reads^26,27^, but they are organized around aggregate signal tracks. Two regulatory elements accessible on half of molecules give the same aggregate profile whether they open together or mutually exclusively. Distinguishing these cases requires grouping molecules by accessibility or footprint pattern, relating the groups to haplotype, CpG methylation or structural variation on the same molecules, and comparing them across samples. General-purpose browsers offer little support for these analyses, do not display chromatin within sequence absent from the reference, and perform poorly with deep targeted datasets of thousands of multi-kilobase reads at one locus. Dedicated packages plot methylation or occupancy on individual molecules^28–31^, but they are largely restricted to single assays and static views of selected loci.

Here we address both gaps. First, we generalize FiberHMM, our previously introduced footprint caller for Hia5 Fiber-seq^32^, into a framework that calls nucleosomes, accessible patches and subnucleosomal footprints for four chemistry–platform combinations. It models the informative bases of each assay, including the deamination that SsDddA introduces at specific, rotationally phased positions within nucleosomal DNA. For single-cell DAF-seq generated with SsDddA^15,33^, FiberHMM reconstructs both strands of individual fibers so that the assay can be calibrated directly in chromatin, and it infers CpG island methylation on the same molecules. Second, we build FiberBrowser, a genome browser and analysis platform that treats each read as an individual chromatin molecule. It reads FiberHMM’s annotations directly and combines interactive clustering of molecular states, allele-specific footprinting and inspection of non-reference structural variants with reproducible graphical and Python workflows, so that footprint calls can be queried and compared in real time across molecules, haplotypes and samples.

## Results

### FiberHMM calls nucleosomes and footprints across single-molecule assays

FiberHMM (fiberhmm-call) identifies nucleosomes, accessible modification-sensitive patches (MSPs) and subnucleosomal footprints on each molecule of an aligned BAM, giving every assay one per-molecule representation (Fig. 1a). It writes these calls, with their evidence scores, back to the BAM as Molecular Annotation (MA) tags (https://github.com/fiberseq/Molecular-annotation-spec; Supplementary Table 1). The FiberBrowser interface and Fiber-seq inference of regulatory elements (FIRE)^20^ read this representation directly, without format conversion.

FiberHMM represents each molecule by the sites where the assay can report a modification and the modifications observed there. The assays differ markedly in both quantities (Fig. 1b and Supplementary Fig. 1a,b). PacBio Hia5 Fiber-seq reports about 50 possible sites per 100 bp as it is capable of identifying modified adenines on both strands, roughly twice the resolution of methods that only mark a specific base on one strand, such as targeted cytidine deaminase-based methods or Fiber-seq sequenced using simplex ONT. Specifically, in Fiber-seq sequenced using simplex ONT, stretches of DNA longer than 10 bp can appear unmodified simply due to an absence of adenine bases. Thus, processing of these data must take into account multiple features that are common to all assays, as well as features unique to a specific assay (Fig. 1a). To accomplish this, FiberHMM employs a two-state HMM that segments each molecule into protected domains and accessible MSPs. Two log-likelihood-ratio (LLR) refinement steps then split protected domains at internucleosomal linkers and call subnucleosomal footprints, scoring each interval against the probability that its informative sites would have been modified if it were accessible. Refinement splits long HMM-protected domains into individual nucleosomes and calls short footprints between them where the data support protection (Fig. 1a).

To select a default LLR threshold, we simulated protected spans of 8–60 bp in motif-centered genomic sequences, with matched fully accessible controls, from each assay’s accessible- and protected-state probabilities (Supplementary Fig. 2a,b,d). The threshold trades sensitivity against specificity, because lower values recover more short footprints but also call more footprints in accessible DNA (Supplementary Fig. 2a,b). We use LLR 5, the lowest integer threshold at which calls in accessible controls fell below 2% for every chemistry, including SsDddA. Users can change it with fiberhmm-call --min-llr. In held-out naked DNA, calls at LLR 5 covered 3.6% of fixed windows, mostly short calls on under-labeled molecules (Supplementary Fig. 2g). At this threshold, recovery follows each assay’s information density. PacBio Hia5 recovered 91% of 10-bp targets, DddB recovered 87% of 40-bp targets, and single-strand ONT Hia5 recovered 26% even at 50 bp.

The same pattern appears in endogenous calls (Fig. 1c). PacBio Hia5 yields abundant footprints below 20 bp and a sharp mononucleosome peak near 145 bp which closely matches the fibertools nucleosome caller on the same molecules. DddB and ONT Hia5 in *Drosophila* embryos yield fewer short footprints and broader nucleosome peaks, including more dinucleosome-sized intervals where linker evidence is sparse. In DddB and PacBio Hia5, refinement recovers short footprints that the HMM merged into protected domains, whereas in sparse ONT Hia5 data it removes short HMM segments that lack support. FIRE run on FiberHMM segmentation reproduces FIRE on the fibertools segmentation of the same reads, reaching 95–98% directional concordance among the most confident calls (Supplementary Fig. 1e,f), so existing pipelines can use the calls directly. FiberHMM calls also quantify transcription factor binding. At 512 accessible CTCF motifs in each of five cell lines, the fraction of molecules with a footprint covering the complete motif separated ChIP-supported^34^ from unsupported motifs as well as fibertools footprint calls did (AUROC 0.83–0.87 and 0.81–0.88; Supplementary Fig. 2e,f and Supplementary Note 2).

### Rotational refinement resolves SsDddA nucleosomes

Hia5 and DddB fit this framework directly, with emission probabilities estimated from naked-DNA and untreated controls. SsDddA, used in single-cell DAF-seq (scDAF-seq), required two extensions. First, SsDddA deaminates not only accessible DNA but also specific positions within nucleosomes. Distances between deaminations on the same molecule, and deamination rates around inferred dyads, both oscillate with a 10.3-bp period (Fig. 2a). This matches the helical repeat of DNA on the histone surface and resembles the rotational pattern of nuclease cleavage of nucleosomal DNA^35,36^. The baseline HMM reads these internal events as linkers and breaks nucleosomes into fragments (Fig. 2b). FiberHMM instead identifies nucleosome cores with relaxed emission probabilities and keeps deaminations that fit the rotational phase inside the particle (fiberhmm-call --mode ddda). On the same molecule this yields individual, mononucleosome-sized calls while preserving the accessible flanks (Fig. 2b). Across 20,915 molecules, the phase model doubles the number of called intervals per kilobase (Fig. 2c and Supplementary Fig. 3a,b). Most of the gain comes from long merged intervals being resolved into separate particles. Most nucleosomes carry at least one internal deamination, often on one side of the dyad, and the internal deamination rate varies with nucleosome position across the *NAPA* and *UBA1* amplicons (Supplementary Fig. 3c–e). DddB showed little internal deamination at the enzyme concentrations used, so its nucleosomes are called with the standard refiner. Because adjacent same-strand SsDddA targets convert together, FiberHMM counts each such run once (Supplementary Fig. 3f–h).

### Duplex calibration and CpG methylation inference enable SsDddA footprinting

Calling footprints in SsDddA data requires both estimating deamination probabilities without a naked-DNA control and accounting for CpG methylation, which has been shown to have a modest effect on SsDddA activity^15,16^. SsDddA has no usable naked-DNA control, because fully accessible DNA accumulates too many uridines to amplify. We therefore calibrated accessible- and protected-state probabilities in chromatin using physical duplexes from scDAF-seq data, in which the separately amplified C-to-T and G-to-A strands of one fiber are both sequenced (Fig. 2d). Because the two strands are deaminated and read independently, an interval called accessible or protected on one strand provides a fixed window in which to measure deamination on its partner. Strands can be readily paired using heterozygous variants that distinguish DNA arising from either of the two haplotypes within a single cell. However, without chaining reads together, sequence alone could not decide the mate for many duplexes. To address this, we developed a tool within FiberHMM that pairs the strands by their nucleosome patterns, using deamination-insensitive sequence differences where they exist (fiberhmm-pair; Supplementary Fig. 4a–c), enabling pattern-based pairing to recover duplexes that haplotype and sequence cannot assign. Where the two homologs differ in sequence, so that sequence can identify the mate independently, pattern-based pairing agreed with it in 96% of cases (Supplementary Fig. 4c). In windows called accessible on one strand, 89% of partner-strand sites were deaminated, compared with 10% in windows called protected (Fig. 2e). The contrast is consistent across 12 libraries and whichever strand serves as the query, and estimates from Hia5-defined accessible regions are broadly consistent (Supplementary Fig. 1c,d). Calling both strands together also fills each strand’s gaps with its partner’s informative sites, more than doubling recovery of short footprints (Supplementary Fig. 4d).

SsDddA deamination is mildly to moderately reduced by CpG methylation^15,16^, so methylated CpGs tend to remain undeaminated even when they are not protein bound. Although this effect is too weak to call methylation at single CpGs, we reasoned that it could classify entire CpG islands as methylated or unmethylated directly from DAF-seq data. FiberHMM first segments each molecule with all CpGs masked and then compares CpG with non-CpG deamination within the called MSPs of each island (fiberhmm-tag-m5c). For each molecule, the deamination rate at non-CpG cytosines within those MSPs sets an accessible-state baseline, and the CpG outcomes are scored against calibrated methylated and unmethylated emission models with equal prior odds. Across one 890-bp island, for example, 32 of 55 non-CpG cytosines were deaminated but only 1 of 15 CpGs, classifying the island as methylated, in agreement with independent long-read methylation (Fig. 2f,g). Genome-wide, unmethylated island calls agreed with long-read CpG methylation at 99.9% precision (Online Methods). For footprint calling, FiberHMM therefore treats CpGs as methylated by default and uses their deamination evidence only within confidently unmethylated islands, which prevents undeaminated methylated CpGs from being read as protection. Differences in nucleosome density between methylated and unmethylated islands likely contribute to this classifier, but reduced deamination at methylated CpGs also contributes directly: CpG-specific loss persisted relative to neighboring non-CpG cytosines of matched accessibility and across MSP lengths and nucleosome geometries (Supplementary Fig. 5e–g and Online Methods).

### FiberBrowser turns single-molecule visualization into real-time population analysis

Conventional genome browsers are built around aggregate tracks that obscure single-molecule heterogeneity, and they display molecules without analyzing them. We therefore built FiberBrowser, an interactive visual and computational platform organized around individual chromatin fibers rather than population averages (Fig. 3a). Each dataset opens as a panel, synchronized with the others or navigated independently, in which every molecule is drawn as a row of its FiberHMM calls, with nucleosomes in green, footprints in sky blue and accessible DNA left pale (Fig. 3a, labels 2, 4 and 7). Shared annotation tracks and metaprofiles of nucleosome and footprint occupancy sit above the molecules (Fig. 3a, labels 5 and 6), and raw modifications, CpG methylation and other layers can be overlaid on the same rows. FiberBrowser’s toolbar and analysis sidebar sort, filter and cluster molecules and scan for peaks and variants, and cluster assignments are drawn beside the molecules (Fig. 3a, labels 3 and 8–10). Sessions are opened, saved and exported from the file controls (Fig. 3a, label 1).

To combine responsive display with quantitative analysis, FiberBrowser separates the rendering of the visualized reads from background computation. The view draws a sample of molecules for speed, while sorting, filtering, clustering and co-occupancy analyses use every eligible molecule in the region. Molecules from several datasets can be jointly clustered or sorted by their accessibility or footprint patterns over selected elements, showing at once how chromatin states are distributed across samples. At *NANOG*, joint clustering of GM12878, H9 and H1 Fiber-seq on accessibility at the promoter and a nearby enhancer separates three states (Fig. 3b). One state is closed at both elements and contains almost all lymphoblastoid GM12878 molecules. The two embryonic stem cell lines instead spread across all three states, with most of their molecules in the two accessible states, which differ in which elements are open. The cluster strip, per-cluster metaprofiles and composition chart display these states, and every summary can be traced back to the calls on individual molecules.

Every analysis is saved with the session, its results can be exported, and every function can be run through a Python API for scripted or headless use. The interface also includes a searchable data library with automatic quality control and tools for targeted assays, such as amplicon primer design (Supplementary Fig. 6). Displaying molecules is comparable to IGV desktop on a first visit and faster on revisits, and it remains possible at depths where igv.js runs out of memory. Unlike IGV, FiberBrowser also analyzes every molecule in the region. At a 3,000-molecule amplicon, sorting, clustering and footprint co-occupancy of all molecules each take one to two seconds (Supplementary Fig. 7a,b; Supplementary Note 1 and Supplementary Tables 2–5).

### Dissecting allelic footprints, DNA methylation and non-reference structural variants on single fibers

A defining advantage of long-read single-molecule assays is that each read captures genetic variants, CpG methylation and chromatin footprints on the same DNA molecule. In FiberBrowser, molecules can be split by haplotype or allele, colored by methylation or grouped by a structural variant, then clustered and compared within seconds. At the imprinted *SNRPN* promoter in H9 embryonic stem cells, phasing molecules by heterozygous SNPs and overlaying CpG methylation shows the imprint directly on single molecules (Fig. 4a). HP1 molecules carry a broad, largely unmethylated accessible region across the differentially methylated region, whereas HP2 molecules are densely methylated and packed with nucleosomes. Joint clustering placed 30 of 33 HP1 molecules but only 6 of 33 HP2 molecules in the accessible state, matching the parental imprint. The biallelic *RPLP0* promoter, by contrast, looked alike on both haplotypes.

Beyond the accessibility of whole elements, FiberBrowser resolves how single-nucleotide variants change individual binding events, even when promoter accessibility is unchanged. In GM12878, a heterozygous C>A variant falls in the core of an NRF1 motif at the bidirectional *SERINC1–PKIB* promoter. FiberBrowser’s variant scan finds the variant in the reads and splits the molecules by the base they carry (Fig. 4b). The promoter is open and largely unmethylated on both alleles, but only the reference allele is bound. A footprint covers the motif on 51 of 59 molecules carrying C and on 4 of 73 carrying A, while the rest of the promoter looks alike. The variant therefore abolishes occupancy at that NRF1 element without changing promoter accessibility or CpG methylation. Both H1 libraries heterozygous for the same variant show the same allelic footprint.

FiberBrowser also reveals chromatin inside structural variants that reference-based browsers cannot show. When molecules carry an insertion absent from the reference genome, standard browsers mark or clip the inserted sequence and hide its chromatin. FiberBrowser instead builds the inserted allele from the carrier molecules and draws their chromatin calls across it. In H1, a 7.5-kb insertion lies on one haplotype (Fig. 4c). On the reference genome, carrier molecules show regular nucleosome arrays on either side of the insertion. Expanding the inserted sequence reveals a 156-bp nucleosome-free region on most carrier molecules, with a footprint at its center on about 80% of them. The footprint contains a canonical CTCF motif, so the insertion brings a CTCF site, footprinted on most molecules, that analyses on the reference genome miss entirely.

### Footprint representations transfer cell-type classifiers across assays

Because FiberHMM translates diverse chemistries into a common representation of nucleosomes and footprints, models trained on one assay should also interpret another. We trained a convolutional neural network (CNN) on footprints and reference sequence in 5-kb promoter windows to distinguish five Hia5 Fiber-seq cell lines (GM12878, a lymphoblastoid cell line (LCL); H1; H9; K562; HepG2), combining promoter-level evidence into one prediction (Fig. 5a and Supplementary Fig. 8a–c). The CNN classified all 40 held-out Fiber-seq pseudo-cells correctly, each built from one or two molecules per promoter (Fig. 5b). Applied without retraining to a different assay and enzyme, it identified all 12 HG002 scDAF-seq cells as LCL (Fig. 5b). Downsampling the scDAF-seq molecules showed how assignment depends on the number of molecules (Fig. 5c). Assignment stayed above 99% with 25% of molecules and above 50% with 0.2%. With one or two molecules per cell it fell to near chance (24%, versus 20%), so the predictions follow the information in the molecules rather than a default class.

By contrast, a CNN with the same architecture trained on raw m6A patterns did not transfer and took significantly longer to train. It also classified all 40 Fiber-seq pseudo-cells correctly, but it assigned none of the scDAF-seq cells to LCL (Fig. 5b; Supplementary Fig. 8g). Footprint inference therefore makes trained models portable across single-molecule assays. A masked-token transformer trained only on Hia5 Fiber-seq transferred in the same way. It predicted masked nucleosome, MSP and footprint labels in scDAF-seq with 98.2% balanced accuracy, compared with 73.4% for a first-order Markov baseline (Supplementary Fig. 8d–f,h).

## Discussion

Our study presents a unified framework for the analysis and interactive exploration of diverse single-molecule chromatin assays, FiberBrowser and its analytical engine, FiberHMM. Across distinct enzymes, chemistries and sequencing platforms, FiberHMM translates heterogeneous modification readouts into a common per-molecule representation of nucleosomes, accessible DNA and subnucleosomal footprints. FiberBrowser then allows thousands of molecules to be clustered, phased and compared in real time while retaining access to the observations underlying every call.

This approach preserves the resolution supported by each experiment. Because footprint evidence weighs observed modifications against the informative sites where the enzyme could act, FiberHMM distinguishes limited information from evidence of protection. Dense PacBio Hia5 Fiber-seq recovers transcription factor footprints below 20 bp, whereas lower-resolution, single-stranded ONT Hia5 Fiber-seq is better suited to nucleosome architecture. Footprint discovery requires no motif annotations, factor identities or prespecified footprint widths, although assigning a protein to an inferred footprint still requires independent evidence such as motifs or ChIP. Existing tools serve complementary roles. Fibertools calls m6A and supports broad manipulation and analysis of Fiber-seq data, IDLI models nucleosomal substructures in SAMOSA, and HiddenFoot evaluates binding at supplied sites. By contrast, FiberHMM returns per-molecule intervals for multiple chemistries in one BAM-native format, and its output can substitute into chemistry-specific pipelines, as shown for FIRE.

Combining this footprint caller with FiberBrowser reveals regulatory mechanisms that aggregate tracks and reference-bound browsers miss. Because long reads preserve genetic variants, CpG methylation and chromatin structure on the same molecules, splitting and clustering molecules links genotype directly to chromatin state. The examples here include the imprinted alleles at *SNRPN*, an NRF1 site lost from an open promoter through a single base change, and a CTCF site carried by a 7.5-kb non-reference insertion.

The transfer of cell-identity and masked-token predictions from Fiber-seq to scDAF-seq shows that this shared representation retains information about chromatin state across chemistries. A classifier trained on Fiber-seq footprints recognizes lymphoblastoid identity in single-cell DAF-seq without retraining, whereas a model trained on raw modifications fails to transfer. Additional scDAF-seq cell types are needed to establish the specificity and generality of transfer. Deeply sampled reference maps in one assay could then help interpret sparse single-cell measurements from another, allowing assays with distinct advantages to contribute to a common analysis of chromatin state.

We designed FiberHMM and FiberBrowser to accommodate new single-molecule chemistries as they emerge. For conventional methyltransferases and deaminases, low-coverage treated naked-DNA and untreated controls suffice to calibrate the observation model. SsDddA shows how rotational phase modeling, physical-duplex pairing and CpG island inference can extend the framework when naked-DNA controls cannot be amplified and enzyme activity depends on DNA methylation. Improvements in ONT m6A detection could be incorporated by updating the corresponding model parameters, and extension to EcoGII, the SMF methyltransferases and other enzymes awaits calibration and validation.

FiberBrowser and FiberHMM are publicly available on GitHub, and we welcome collaborations to support additional single-molecule approaches.

## Online Methods

### Software implementation and supported assays

FiberHMM v3.0, the analytical engine of FiberBrowser, provides command-line workflows for calling and duplex reconstruction. The FiberHMM command line and the FiberBrowser interface invoke the same engine, so interactive use and batch analysis share the fitted model and numerical implementation. Standard BAM outputs retain molecule identities, raw observations, calls, derived annotations and provenance.

FiberHMM is implemented in Python and processes aligned long-read BAM files^37^ through the command-line entry point fiberhmm-call. SsDddA, DddB and Hia5 are selected with assay presets, and Hia5 input is additionally designated as PacBio or ONT. Coordinate-sorted, indexed BAMs are processed in genomic chunks and merged without changing read names or source alignments; unindexed input can be streamed. Secondary, supplementary and unmapped records are excluded from inference. Analyses used FiberHMM 3.0.0 and FiberBrowser 3.0.0; the exact source commit and emission-table hash for each analysis are recorded in the accompanying provenance manifests.

Performance was measured on a desktop workstation. On a 4-Mb H1 Hia5 PacBio BAM containing 19,540 primary records, FiberHMM completed calling in 163 s with 218 MiB peak memory using eight compute threads. Browser display, navigation and analysis benchmarks are described in Supplementary Note 1 and Supplementary Fig. 7a,b.

### Molecular Annotation encoding and FIRE interoperability

FiberHMM writes, and the FiberBrowser interface reads, the Molecular Annotation specification developed by Mitchell R. Vollger (https://github.com/fiberseq/Molecular-annotation-spec). The MA:Z tag stores the original read length and 1-based start-length intervals in original molecule orientation, together with annotation type, biological strand and zero or more typed quality declarations. Q declares a linear 0–255 byte and P a Phred-scaled byte. AQ:B:C stores the declared bytes in MA interval order, and AN:Z stores optional names aligned to all MA intervals. Reference coordinates are derived through the alignment and do not replace molecule coordinates.

FiberHMM layers use nuc.Q or nuc.QQQ for nucleosome quality and optional edge qualities, msp. for accessible-patch coordinates and tf.QQQ for footprint TQ and left/right edge qualities, where TQ = min(255, round(10 × LLR)). Final MSP coordinates are also mirrored in the fibertools as/al arrays so that existing fibertools and FIRE versions can consume the same segmentation.

For FIRE interoperability, fibertools-rs 0.13.0 ft fire was run separately on fibertools and FiberHMM segmentations of the same 10,000-read GM12878 PacBio subset; 9,781 reads carried FIRE annotations in both outputs, and final FiberHMM MSP intervals matched the as/al arrays consumed by FIRE. Positive FIRE calls followed the documented aq > 230 definition, and a directional call was shared when it overlapped a positive call from the other segmentation by at least 1 bp on the same read. At aq > 230, approximately 81% of fibertools calls and 79% of FiberHMM calls were shared. The concordance curve retained calls at each cumulative minimum aq; at aq ≥ 249 (aq/255 ≥ 0.976 estimated precision) the directional fractions were 98.0% and 94.8% (95% molecule-bootstrap intervals 97.6–98.3% and 94.3–95.4%). The 17,340 shared overlap components containing exactly one call from each segmentation had a median absolute width difference of 8 bp (interquartile range 4–21 bp; Supplementary Fig. 1e,f).

### Molecular observations and informative sites

FiberHMM represents a molecule by its mapped reference blocks, the sequence positions at which the selected enzyme could generate an observable event (informative sites) and the subset of those positions carrying an event. SsDddA and DddB informative sites were reconstructed on the relevant cytosine strand after alignment to the reference. Hia5 informative sites were adenines or thymines in the reference frame, with platform-specific modification tags supplying observed m6A. Aligned deletions and uncovered bases were treated as missing observations, not as evidence of protection.

Emission tables specify the probability of an event and of no event in the accessible and protected states for each encoded sequence context. Hia5 (PacBio and ONT) emissions and DddB protected-state emissions were derived from assay-matched controls; DddB accessible-state emissions were recalibrated in vivo, as described below. PacBio Hia5 emissions were counted in seven-base contexts from Hia5-treated and untreated *Drosophila* S2 genomic DNA, with m6A taken from the PacBio modification calls at ML ≥ 128; initial-state and transition probabilities are those of the original FiberHMM Hia5 PacBio model^32^. Fully accessible naked-DNA SsDddA controls accumulate too many uridines to be amplified and sequenced efficiently, whereas nucleosomes and bound proteins keep conversion in chromatin within the recoverable range. The SsDddA nucleosome and footprint components therefore use separate tables: the nucleosome model accounts for internal rotational deamination, and the subnucleosomal table was derived from reconstructed physical duplexes as described below.

Adjacent same-strand SsDddA targets (CC on C-to-T reads, GG on G-to-A reads) do not convert independently. At confidently accessible *NAPA* sites, the second target of an isolated adjacent pair converted in 93% (CT) and 93% (GA) of molecules when the first did, and in 3% and 5% when it did not; targets 2–6 bp apart were not positively coupled (Supplementary Fig. 3f,g). Because the emission model treats each target as an independent observation, such a run would count one event more than once. FiberHMM therefore keeps only the 5′-most target of each run of adjacent same-strand SsDddA targets by default, on every informative-site set it evaluates: calling, footprint recall and physical-duplex merging (keep-one thinning, --daf-mask-runs; DddB informative-site sets are not thinned). This raised the precision of footprint calls read on the complementary strand of HG002 physical duplexes (Supplementary Fig. 3h).

### DddB emission calibration

DddB ONT emissions were first estimated from dm6-aligned *Drosophila* naked-DNA controls: a whole-genome-amplified (WGA), DddB-treated control and an unamplified control library containing untreated and non-WGA deaminated molecules. For each primary alignment, combined conversion was defined as (C→T + G→A)/(reference C + reference G). Analyses required mapping quality ≥20 and an aligned span ≥1 kb. The WGA control contained a prominent under-deaminated population. For a read with k conversions among n combined C/G informative sites, strand-adjusted conversion efficiency was estimated as the Jeffreys-smoothed quantity (2k + 1)/(n + 2). A two-component Gaussian mixture fitted after logit transformation to 395,658 eligible WGA molecules placed the equal-posterior boundary at 17.9% combined conversion (19.8–19.9% for unsmoothed logit- and log-rate fits), and WGA molecules with ≥20% combined conversion were retained as the accessible control.

For the unamplified control, the corresponding mixture fitted to 500,000 eligible molecules separated a low-conversion component centered at 1.03% from a deaminated component centered at 25.5%. Molecules with <1.75% combined conversion, a threshold more stringent than the 2.14% boundary for 95% posterior assignment to the low-conversion component at the median informative-site count, were retained (30.3% of eligible molecules) as a conservative protected-state background.

Up to 100,000 passing reads per control were used for emission estimation, excluding 10 bases from each read end. C→T observations were counted in C-centered seven-base contexts, and G→A observations were reverse-complemented into the same context space. The fixed central C leaves six variable flanking bases, giving 4,096 contexts. These control-derived probabilities replaced only the emission matrix; initial-state and transition probabilities were copied unchanged from the PacBio Hia5 model.

Comparison with in vivo data indicated that the naked-DNA table underestimates the accessible-state deamination rate in chromatin. The accessible-state emissions were therefore recalibrated against two in vivo matched Hia5 Fiber-seq, which set the overall expected modification burden, and a whole-genome DddB DAF-seq dataset, which set the dependence on sequence context.

At four *Drosophila* amplicons (*chrb*, *hb*, *scyl* and *ind*), each enzyme’s event rate inside long accessible regions (MSPs of at least 150 bp, with 15 bp trimmed from each edge) was compared with the rate predicted by its own emission table over the same sites, using wild-type 2–4-h DddB DAF-seq and 2–4-h PacBio Hia5 Fiber-seq. Hia5 reached 0.56–0.61 of its table-predicted rate, reflecting occupancy by bound factors and partial protection, whereas DddB reached 0.82–0.93 of the naked-DNA prediction. Relative to Hia5, DddB therefore exceeded the naked-DNA table 1.36–1.56-fold (median 1.45), or 1.20–1.41-fold (mean 1.29) when sites within called footprints were excluded, and the accessible-state level was set to 1.29 times the naked-DNA table. An independent per-context fit, estimating each enzyme’s accessible-state rate against accessibility measured on a separate half of its own molecules, gave a Hia5-calibrated level of 1.67-fold (95% locus-bootstrap interval 1.58–1.75; Supplementary Fig. 1a); the more conservative 1.29-fold was retained. This fit used five-base contexts at the *hb*, *ind*, *chrb* and *scyl* amplicons in wild-type 2–4-h embryos (DddB called with the naked-DNA table; Hia5 PacBio 2–4 h), modeled each context as P(event) = a·p_acc_ + (1 − a)·p_prot_ with a the fraction of molecules from the other half whose MSP covers the site outside called footprints, and gave protected-state rates of 0.0022 (DddB) and approximately 0 (Hia5); excluding the 43 DddB contexts (14% of sites) fitted at the upper bound gave 1.65-fold. Deamination inside called nucleosomes was low and showed no rotational periodicity, so protected-state emissions were left unchanged.

Context dependence was estimated from a whole-genome *Drosophila* DddB DAF-seq nanopore dataset, using a fixed-seed 25% subsample (938,265 molecules; 958 million target informative sites; at least 50,000 informative sites for every six-base context). Relative to the naked-DNA table, deamination in chromatin depended on the bases immediately flanking the target C. These were fitted as additive effects on the logit scale by ridge-penalized Poisson regression (penalty 0.5), relative to an adenine neighbor: 5′ C −0.32, T +0.45, G −0.34; 3′ C −0.31, T −0.74, G +0.37. A single multiplicative scale did not capture this pattern. Corrections that included more distant flanking bases fitted the whole-genome data slightly better but agreed less well with the amplicon data, which were not used for fitting, so only the adjacent bases were retained.

The resulting accessible-state emissions are logit p_acc_(c) = logit p_acc,naked_(c) + α + β_5′_(c) + β_3′_(c), with α = 0.845 chosen so that the informative-site-weighted mean accessible-state probability on the amplicons is 1.29 times the naked-DNA value (0.409 to 0.527), and capped at 0.95 (170 of 4,096 contexts). Per-context values changed 0.80- to 2.18-fold (median 1.26). Initial-state and transition probabilities were unchanged. This in vivo-calibrated table is the default DddB model.

### Hia5 ONT emission calibration

Hia5 ONT emissions were estimated from two control libraries of *Drosophila yw* 2–4-h embryo genomic DNA, sequenced on a PromethION (R10.4.1 flow cells): naked DNA treated with Hia5 for the accessible state and untreated DNA for the protected state. Reads were basecalled with dorado 2.0.1 (sup v5.2.0 with the 6mA v1 modified-base model) and aligned to dm6 with minimap2^38^ (map-ont). Primary alignments with mapping quality ≥20 and an aligned span ≥1 kb were used, and m6A calls required ML ≥248, the threshold used for ONT Hia5 calling. The naked-DNA control contained a low-methylation population. A two-component Gaussian mixture fitted to the logit-transformed, Jeffreys-smoothed per-read fraction of base-called adenines called m6A placed the boundary for ≥0.95 posterior assignment to the methylated component at 11.4%, and the 49,961 of 62,033 eligible reads above it were retained. All 842,434 eligible untreated reads were used. Events and non-events were counted in adenine-centered seven-base contexts in the sequenced-strand orientation, excluding 10 bases at each read end (4,096 contexts per state; 1.97 × 10^9^ protected-state positions). Initial-state and transition probabilities were copied from the PacBio Hia5 model.

### First-pass hidden Markov segmentation

The first stage is the two-state FiberHMM introduced previously^32^. The states represent accessible and protected sequence, and emissions are selected by assay and local sequence context. The model is run over the ordered informative-site set of each molecule and projected back to reference coordinates. Contiguous accessible segments define modification-sensitive patches (MSPs), and protected segments provide the initial nucleosome-scale topology. The generalized method retains the published HMM parameters and topology and adds observation presets, post-HMM refinement and a common output format.

### Nucleosome and subnucleosomal-footprint likelihood refinement

The subnucleosomal step is referred to as footprint refinement; software commands and figure labels abbreviate it as TF refinement or TF recall.

Post-HMM inference is divided into nucleosome and subnucleosomal-footprint tasks. For a candidate interval with observations xi and sequence contexts ci, FiberHMM accumulates the evidence

$$LLR=\Sigma_{i}\;log\left[P\left(x_{i}|\right.\right.protected,\;\left.c_{i}\right)/P\left(x_{i}|\right.\;accessible,\;\left.\left.c_{i}\right)\right].$$

For Hia5 and DddB, the two refiners use this context-specific additive score in opposite directions. Within each HMM-protected interval, the sign-inverted score identifies local runs of accessible evidence that support linker cuts; the protected-state score is then applied to each remaining fragment to define conservative nucleosome edges and an interval quality score. The resulting accessible sequence is merged with the original HMM-accessible state to rederive MSPs. The footprint refiner then selects disjoint intervals within the scan domains (MSPs and short HMM-protected candidates) that maximize the sum of interval LLRs minus a fixed penalty per selected interval, subject to a minimum informative-site requirement; this multi-interval selection is exact. SsDddA replaces the accessible-cut step with the rotational refinement described below.

The default footprint threshold is LLR 5 for SsDddA, DddB, PacBio Hia5 and ONT Hia5, with at least three informative sites. Each interval stores TQ = min(255, round(10 × LLR)), so the default TQ floor is 50 in every preset. TQ can be used for downstream filtering, but changing the interval penalty requires fresh decoding because it can split, join or newly detect intervals. TQ is an evidence score, not a posterior binding probability or false-discovery rate.

For the threshold benchmark in Supplementary Figure 2a,b,d, 320 motif-centered genomic contexts were selected, the motif-forward and reverse-complement informative-site sets were evaluated independently, and protected spans of 8, 10, 15, 20, 25, 30, 40, 50 and 60 bp were programmed. Sixteen protected draws and sixteen matched fully accessible controls were generated for every context, orientation and size, giving 92,160 protected and 92,160 accessible-control trials per chemistry and threshold. No feasibility or minimum-information filter was applied before evaluation. Each threshold from LLR 2 through 8 was rerun, and a trial was recovered when a returned subnucleosomal interval covered the programmed center. SsDddA used the full default HMM, rotational nucleosome refiner and footprint refiner, with the two strand-specific informative-site sets called independently; the other assays used their default calling settings. The endpoint measures center-covering footprint recovery rather than boundary accuracy. Uncertainty bands are 95% intervals from resampling genomic context blocks. The selected common setting, LLR 5, is the first integer threshold at which simulated accessible-control calls were below 2% in all four chemistries. Empirical false calls were measured in the PacBio Hia5 naked-DNA control library (*Drosophila* S2 genomic DNA treated with Hia5), the library used to fit the accessible-state emissions. The 46,678 molecules used for emission fitting were excluded, and 20,000 of the remaining primary alignments (MAPQ ≥20, aligned length ≥1 kb) were sampled with a fixed seed and called with the default PacBio Hia5 settings at LLR 5. One 800-bp window was fixed per molecule, and a false call was scored when a footprint covered the window center, the endpoint used for the simulated accessible controls. The model expectation was obtained by drawing observations from the accessible-state emissions at each molecule’s own informative sites and calling them in the same way (mean of three repeats). Per-molecule labelling was the fraction of informative A/T sites called as m6A. To separate variation in labelling between molecules from effects within molecules, the simulation was repeated with each molecule’s accessible-state probabilities scaled to its observed labelling, which gave 1.5% (95% CI 1.4–1.5%). Intervals are percentiles of 5,000 molecule bootstrap resamples (Supplementary Fig. 2g).

### Rotational SsDddA nucleosome refinement

SsDddA nucleosome refinement begins from permissive HMM-protected cores. Candidate extensions are evaluated against a phase model, distributed with FiberHMM, describing the probability of internal deamination as a function of distance and rotational phase from the protected core. The model allows a periodic series to skip one or more expected events and permits modest variation in spacing. A proposed edge is supported when accessible evidence in the flank is inconsistent with continuation of the periodic series; boundary uncertainty is retained when competing extensions have similar support.

The radial deamination profile of the phase model was estimated from pooled *NAPA* and *UBA1* amplicon molecules and applied unchanged to the genome-wide scDAF-seq libraries, which were not used for fitting; *NAPA* and *UBA1* diagnostics therefore overlap the fitting data. The molecule-matched ablation disabled only the phase model, with code, input molecules and all other parameters identical (Supplementary Fig. 3a,b). Internal-event counts, sidedness and caller-independent recurrent-site maps were evaluated as separate diagnostics (Supplementary Fig. 3c–e). DddB nucleosomes showed no detectable rotational phase, and the standard likelihood refiner remains the default.

Nucleosome density in the phase-model ablation was calculated by dividing the number of called intervals by the sum of CIGAR-aligned bases over the same primary alignments. Length categories were defined before counting as less than 120 bp, 120–200 bp, 201–249 bp and at least 250 bp. The two call sets contained identical molecule names and 64,022,684 mapped bases.

The molecule-matched ablation included 20,915 scDAF-seq molecules. In it, 120–200-bp intervals increased from 1.02 to 3.72 per mapped kb, intervals of at least 250 bp became uncommon, and total called intervals increased from 2.26 to 4.60 per mapped kb. Event sidedness was evaluated against an equal-side finite-count null rather than treating asymmetry among sparsely modified particles as evidence of a preferred side.

### SsDddA event burden at recurrent nucleosome sites

Recurrent nucleosome dyads at *NAPA* and *UBA1* were nominated independently in two deterministic molecule halves. Peaks retained in both halves defined fixed 120-bp windows, and each molecule was evaluated only at centers learned from the opposite half. Every primary alignment spanning at least 95% of a window contributed its observed SsDddA events and non-CpG informative sites. Sequence- and radial informative-site weights were fitted separately by conversion strand in the opposite molecule half, and each fiber’s total internal-event count was allocated among covered positions in proportion to these weights. Burden ratios were calculated in the pooled data and separately for CT and GA channels; the figure reports the pooled estimate with saved molecule-bootstrap 95% intervals. Colors indicate enrichment or depletion at family-wise corrected P < 0.05, with nonsignificant positions gray. A secondary analysis restricted the calculation to windows containing a rotationally refined nucleosome call within 75 bp of the cross-fitted center.

### Endogenous call-length distributions

The *Drosophila* DddB amplicon data covering *hb*, *ind*, *scyl* and *chrb* were generated for this study and sequenced by ONT, as were the *Drosophila* yw 2–4-h embryo Hia5 ONT Fiber-seq data and their naked-DNA and untreated controls.

Figure 1c uses all primary mapped bases and all final interval calls from 22 passing DddB libraries spanning the *hb*, *ind*, *scyl* and *chrb Drosophila* amplicons after footprint-only recall at LLR 5 (86,591 molecules; 542,152,784 aligned bp; 249,873 footprint calls; 758,985 nucleosome calls). The Hia5 PacBio distribution uses 3,000 K562 molecules and the Hia5 ONT distribution uses 11,162 *Drosophila* yw embryo molecules (2–4 h; four 50-kb windows), both at the same LLR 5 footprint threshold. Curves report called base pairs per 100 kb aligned per one-base length bin and are smoothed for display only (Gaussian kernel, s.d. 2 bp). HMM-only distributions were obtained before nucleosome and footprint refinement on the same DddB, Hia5 ONT and Hia5 PacBio molecules, with the original SNP masks and read filters; skipped molecules remained in the aligned-base denominator and contributed no calls.

### Real-molecule stage illustrations

For the Figure 1 stage illustrations, raw observations were passed through the FiberHMM HMM and refinement steps at LLR 5, and intermediate nucleosome boundaries were captured immediately before footprint *refinement.* Candidate windows were screened in 160 *hb* DddB molecules, 1,500 *Drosophila* embryo ONT Hia5 molecules and 160 K562 PacBio molecules, and three 600-bp windows were selected to illustrate a nucleosome split or boundary change and a supported subnucleosomal call. Each stage shows the same informative sites and observations in read coordinates. All three assays use the same refinement settings. Read identifiers, windows, intervals and model hashes accompany the plotting data.

### DAF duplicate handling and variant screening

Amplified DAF reads were grouped into same-strand amplification families using alignment endpoints and deamination fingerprints. Clustering was constrained to compatible endpoints and required a minimum number of informative events; reads that could not be fingerprinted remained labeled as such. The default caller retained all source reads; population analyses used one deterministic representative per family. Potential opposite-conversion variants were screened only when recurrent evidence was observed in both directions; screening was disabled automatically below its coverage requirement and did not modify the HMM or footprint likelihoods.

### Reconstruction of physical scDAF-seq duplexes

Duplexes were reconstructed independently within each scDAF-seq library after collapsing same-strand amplification families (see DAF duplicate handling and variant screening). Reads were classified by their dominant SsDddA conversion channel as CT or GA, and candidate mates were restricted to overlapping reads from opposite channels. Each strand was called independently before pairing. Reference-coordinate nucleosome dyads from the broad call were placed on a 10-bp grid and convolved with a 30-bp Gaussian kernel, producing a nucleosome-scale fingerprint that retains nucleosome order and approximate position while absorbing strand-specific boundary variation.

Candidate pairs shared at least 1.5 kb of genomic span and contained at least four nucleosomes from each strand within that span. Similarity was the maximum normalized cross-correlation between the two nucleosome-density signals across register shifts of up to 60 bp. An assignment required reciprocal selection, correlation above the empirical unrelated-pair floor and a sufficient advantage over the next candidate in both directions. Within the retained pairs, the assigned GA mate scored higher than the best competing GA product for 96.0% of 5,225 CT queries (median scores 0.600 versus 0.255); this margin describes the retained cohort, and independent validation is described below.

Haplotype and deamination-insensitive sequence provided independent constraints. On haplotype-resolved alignments, candidates were restricted to the same maternal or paternal contig. Reads were also compared at shared reference A/T positions, which SsDddA does not alter: a strong reciprocal sequence preference could resolve a pair directly, and gross sequence discordance vetoed a nucleosome-pattern match. Local two-by-two ambiguities were resolved when one diagonal was uniquely supported; unresolved components remained explicitly unpaired. The pairing route, partner, correlation, sequence difference and assignment margin were recorded as BAM tags.

Accepted strands were projected into a common forward reference frame, with CT and GA events, informative-site sets and aligned coverage kept as separate channels; deletions and uncovered positions remained missing observations. Pair identities used for SsDddA calibration were fixed, using only the broad nucleosome architecture, before any query interval was nominated or any model fitted.

The standalone fiberhmm-pair tool additionally supports footprint-only pairing, distinct from the sequence-assisted reconstruction used for the calibration set above. Candidates require at least 1,500 bp of overlap and four nucleosome dyads on each read. A fixed linear score combines lag-tolerant dyad correlation, dyad-anchor agreement, overlap and dyad-count features, and 20-bp-binned non-CpG protection correlations, including a residual relative to the local overlap-component background. A/T agreement, haplotype and footprint LLRs do not enter this score; the reference is used to enumerate enzyme informative sites. A selected edge must be reciprocal-best and exceed each competing edge, or a zero-score null, by a default margin of 1.0; other candidates remain unresolved. BAM provenance selects the calibration appropriate to the nucleosome-calling model recorded in the file. Pair scores, margins, partner identities and model identifiers are retained in BAM tags.

For the genome-wide pairing validation (Supplementary Fig. 4a–c), the fixed footprint score was evaluated across all primary chromosomes in twelve libraries. Connected components overlapping the chromosome 1 pairing-model fitting interval were excluded, and the prespecified chromosome 1 validation interval was retained separately. A/T features were withheld from footprint scoring, and default thresholds were not retuned. Independent sequence relationships classified selected pairs as matching the sequence-supported mate, selecting a different mate, or unresolved. Agreement used only sequence-assessable selections; unresolved relationships were neither successes nor errors. Recovery used all independently sequence-selected pairs, including footprint-unscorable pairs. Confidence intervals used 1,000 Poisson bootstrap resamples of connected components, preserving competing edges. Among the 461,127 selected test pairs, 454,100 (98.48%) had no decisive independent A/T mate assignment, and 77.21% had no homolog comparison. A sequence label was assigned only when both reads had a competing candidate. Where the two homologs are identical in sequence, such a label cannot distinguish the mate from the other homolog’s strand, so agreement was evaluated at loci where both reads mapped uniquely to one HG002 haplotype (diploid remapping): 4,953 of 5,165 selections agreed (95.9%; 95% interval 95.3–96.4%). Same-strand amplification copies were essentially absent from the consensus reads (167 of 10.4 million pairing-eligible reads in copy clusters). These counts describe the availability of independent validation assignments; pairing itself used the fixed nucleosome and non-CpG protection score with sequence and haplotype withheld.

### Joint inference from both strands of a physical duplex

For joint inference, fiberhmm-pair (merge and recall stages) combines an accepted physical pair in forward-reference coordinates while retaining the CT and GA channels and their aligned coverage. Missing flanks and deletions remain unobserved. The HMM, rotational SsDddA nucleosome refiner and footprint refiner run once on the combined molecule using the two-channel observation encoder. The merged record retains both source identities and strand-specific deamination and coverage annotations. Original single-strand intervals remain in the separate source BAMs.

Population analyses count a successfully merged duplex once and retain both source identities. Tagged but unmerged pairs are rejected rather than counted as independent molecules. Merge failures retain the unchanged source records by default; an explicit pairs-only export restricts output to successfully merged molecules.

### Physical-duplex calibration and validation of SsDddA footprint emissions

Calibration used physical duplexes from the first 40 Mb of chromosome 1 in each of the 12 scDAF-seq libraries (23,388 duplexes), excluding CpG sites and the terminal 10 bp of each read. One strand was designated the query and the other, its mate, was used only for evaluation. Query-accessible observations were selected inside the first-pass accessible state from local neighborhoods containing at least ten non-CpG informative sites with complete query deamination. Query-protected intervals were maximal runs of at least nine consecutive missed non-CpG informative sites, bracketed by events and spanning no more than 40 bp; the first and last query misses fixed the interval. Mate observations neither created a candidate nor moved its edges. Both CT-to-GA and GA-to-CT directions were analyzed.

Accessible- and protected-state mate outcomes were fitted separately with additive logistic models using the six flanking bases, with model structure and regularization selected by leave-one-library-out, state-balanced log loss (ridge penalty 1 in the validation fits below). The resulting context-specific probabilities were serialized into the shared FiberHMM model structure and used only by the footprint LLR; the first-pass SsDddA HMM and phase-aware nucleosome model were unchanged.

Several controls tested whether query nomination selected molecules with unusual overall SsDddA exposure. Event fractions outside the nominated interval were calculated across each duplex, and the accessible/protected comparison was repeated on common support. A site-level model included sequence context, library, transfer direction, the exterior event fraction and its interaction with query state, with covariance clustered by physical duplex. Broken-pair controls were matched by library, direction and exterior-event stratum.

For the held-out physical-mate validation reported in Supplementary Figure 2c, 490,934 accepted duplexes across twelve libraries were evaluated, yielding 360,232 query-nominated windows. Eight libraries supplied emission fitting, two were reserved for validation and two for testing; training-library diagnostics excluded the scored library from fitting. Protected and accessible query nominations were fixed before evaluating the mate, with at least three mate informative sites and CpGs excluded. The default interval decoder was rerun at 14 penalties from 0 to 20, rather than filtering scores from a different penalty or using only a maximum fixed-window score. Standard emissions were compared with exact six-base emissions fitted on training libraries. Confidence intervals used 1,000 Poisson bootstrap resamples clustered by physical duplex, preserving repeated windows and both directions. At LLR 5, standard and expanded emissions gave protected-window recovery of 91.73% and 93.52%, respectively, and accessible-window call fractions of 4.14% and 4.28%. Query labels are operational controls, not ground truth for factor binding.

### Alternative SsDddA accessible-rate estimation using Hia5 MSPs

An independent sensitivity analysis used Hia5 accessible segments to nominate SsDddA measurement regions at *NAPA* and *UBA1* (Supplementary Fig. 1c). Hia5 coverage was at least ten, MSP consistency at least 0.7, and recurrent footprint protection was excluded at recurrence 0.05 with at least three supporting molecules. Additional SsDddA protected-run masks required two, three or four consecutive misses, with maximum gap between informative sites 12 bp and span 90 bp. The horizontal reference is the independently reported physical-duplex accessible-mate estimate of 89%, rounded as in Figure 2e.

The genome-wide check processed all 6,022 nonoverlapping 1-Mb cores across 46 HG002 diploid contigs and all twelve scDAF-seq libraries, with Hia5 coverage at least five and MSP support at least 80% (Supplementary Fig. 1d). Recurrent Hia5 footprints were excluded. Both haplotypes of each chromosome remained in the same training, validation or test partition. Each focal SsDddA observation was withheld when selecting accessible neighborhoods: at least six other C/G informative sites within 50 bp had to meet the indicated event-fraction threshold. Confidence intervals resample chromosome groups 2,000 times, retaining both haplotypes together. Context models were fitted on training chromosomes and selected using validation chromosomes; exact six-base models were selected for both strands under the independent Hia5 MSP mask.

Hia5-MSP-based estimates test how SsDddA deamination rates depend on the choice of measurement region (Supplementary Fig. 1c,d). At *NAPA*, masking runs of at least two, three or four missed deaminations gave rates of 90.5%, 84.2% and 80.6%; at *UBA1*, the corresponding rates were 94.0%, 88.4% and 82.6%. Because SsDddA outcomes contribute to the mask, these are selection-dependent rates rather than unbiased conversion probabilities; the independent physical-duplex accessible-mate estimate is approximately 89%.

In the genome-wide HG002 analysis, independent Hia5 MSP selection alone gave test-set event rates of 34.7% and 34.5% for CT and GA. Additional focal-site-excluded neighbor selection raised rates to 86.3–93.3%, depending on the threshold (Supplementary Fig. 1d).

Because neighboring outcomes remain correlated and Hia5 reports a separate population of molecules, these conditional rates corroborate rather than independently establish the duplex-derived accessible-state probability.

### Complete-CpG-island methylation inference

Expanded whole-island validation used per-CpG methylation from HG002 LCL PacBio Fiber-seq (PS00166), a population reference, and all 12 matched scDAF-seq libraries. Methylation was called with pb-CpG-tools v3.0.0 (default settings) after pbmm2 alignment to the HG002 v1.1a assembly and lifted to the HG002 v1.1 diploid reference with per-20-kb offsets; 60,502,765 of 60,511,357 records (99.99%) were placed exactly on a v1.1 CpG, and records that were not were discarded. Islands required at least ten reference CpGs and mean methylation at most 0.10 or at least 0.90 for the extreme-state comparison. Entire chromosome groups, with both haplotypes assigned together, defined fixed training, validation and test sets.

CpG islands were inferred from the indexed reference using 200-bp windows stepped every 10 bp. Windows with GC fraction at least 0.50 and CpG observed/expected at least 0.60^39^ were retained, where observed/expected was the CpG count multiplied by window length and divided by the product of C and G counts; overlapping qualifying windows were merged. Users may instead supply a merged, nonoverlapping BED catalog. The supplied or inferred complete island remains the reporting unit in either case.

Initial MSPs were called with every CpG observation masked in both the first-pass HMM and the refinement steps. For each molecule-island overlap, only CpG and non-CpG SsDddA observations whose query positions lay within an initial FiberHMM MSP were retained. The non-CpG rate across the complete overlap supplied a molecule-specific accessible baseline, adjusted for the calibrated SsDddA 5′ sequence factors. CpG outcomes were summed under the calibrated methylated and unmethylated emission models with equal prior odds. Calls required at least 15 CpGs and 10 non-CpGs and were methylated at posterior at least 0.99, unmethylated at posterior at most 0.01 and otherwise uninformative. No transition or boundary was inferred within an island.

The default fiberhmm-tag-m5c workflow infers islands from the reference, writes the exact catalog on request and records confident methylated and unmethylated islands as MA:ddda_mcg and MA:ddda_ucg spans, respectively. An external --cpg-islands BED overrides the catalog without changing call resolution. The optional audit table retains all three molecule-island states and their evidence counts. SsDddA footprint recall enables CpG masking by default (--use-m5c --cpg-mask-policy unmethylated-only), retaining CpG observations only within confidently unmethylated MA:ddda_ucg spans and excluding all other CpGs from the informative-site set in both the first-pass HMM and refinement. Excluded sites contribute no likelihood, do not count toward minimum evidence, and cannot define footprint boundaries or boundary uncertainty; non-CpG observations retain their ordinary emissions. An alternative setting (--cpg-mask-policy methylated-only) instead excludes CpGs only within MA:ddda_mcg spans.

In a 1-kb regional CpG benchmark against the same reference across 46 haplotype contigs, the truth-window-weighted mean extreme-state AUROC was 0.9941, and mean methylated- and unmethylated-call precision was 90.5% and 79.4% (Supplementary Fig. 5a). This benchmark ranks strongly methylated against strongly unmethylated reference windows. The whole-island classifier, which pools MSP evidence across a complete sequence-defined island without fitting internal boundaries, was evaluated separately. Its 15-CpG evidence requirement was set during development and kept as released; Supplementary Fig. 5c shows agreement and called fraction across evidence floors on validation chromosomes.

In the held-out whole-island evaluation, 25,198 overlaps with truth-unmethylated islands met the requirement of at least 15 informative CpGs and ten non-CpGs. Of these, 22,796 received a confident call (90.5%), and 22,725 agreed with the reference state (99.7%; 95% island-cluster bootstrap interval, 99.6–99.8%). Among 1,630 evidence-eligible overlaps with truth-methylated islands, 898 received a confident call (55.1%), and 872 agreed (97.1%; 95% interval, 94.9–98.9%). Because individual fibers need not match a population reference, the implied false-positive and false-negative rates (0.3% and 2.9%) are upper bounds on molecular calling error. Of confident predictions, 872 of 943 methylated (92.5%) and 22,725 of 22,751 unmethylated (99.9%) agreed with the reference. Footprint calling retains CpGs only in confidently unmethylated islands, so the unmethylated precision determines its accuracy; the lower methylated precision reflects the excess of unmethylated islands in the test set. Intervals were obtained from 1,000 Poisson island-cluster bootstrap resamples, retaining all molecules from each island together.

CpG specificity at matched local accessibility was assessed on the 3,141 evidence-eligible molecule–island overlaps (322 methylated-reference and 2,819 unmethylated-reference) from the test chromosome groups at mapping quality ≥30 across all 12 libraries, using raw aligned observations without restriction to an HMM state. For each focal CpG, local accessibility was the conversion of its ten nearest non-CpG observations within 100 bp, with observations required on both sides; focal non-CpG controls used the same rule with the focal site excluded, so a focal outcome never contributed to its own accessibility estimate. The primary analysis used focal sites with at least eight of ten neighbors converted. CpG and non-CpG conversion were standardized across shared strata of library, strand, 5′ base, local non-CpG activity and non-CpG activity on the same molecule outside the island (at least 20 observations required), retaining 157,048 of 204,248 site observations in 297 strata across 1,051 islands. The difference-in-differences was the methylated-minus-unmethylated change in CpG conversion minus the corresponding change in non-CpG conversion, with 95% intervals from 2,000 island-level bootstrap resamples. The primary estimate was −54.9 percentage points (95% interval −58.5 to −50.9; Supplementary Fig. 5e). Sensitivity analyses varied the neighbor requirement (6/10 and 10/10), radius (50 and 200 bp), mapping quality (≥60), strand, library and omitted test chromosome. Two held-out analyses assigned island state from a deterministic 80% of CpGs and measured conversion at the withheld 20%, using either initial MSPs or raw non-CpG neighbors alone (Supplementary Fig. 5e–g).

Geometry controls used the 3,141 overlaps and their 121,537 CpG and 353,774 non-CpG initial-MSP observations. Methylated-reference islands were more nucleosome-dense than unmethylated-reference islands (median island MSP fraction 0.40 versus 0.49; median linker 36 versus 54 bp), which these stratified controls address. The difference-in-differences was computed by the length of the MSP containing each observation (<100, 100–149, 150–199, 200–299, 300–499, 500–999 and ≥1,000 bp) and by tertiles of island MSP fraction, overlap-weighted MSP length, and median linker length, nucleosome repeat length and nucleosome-covered fraction within ±1 kb. Island state labels were shuffled 10,000 times within the 26 occupied strata formed by linker, MSP-fraction and MSP-length tertiles. Without matching on local accessibility, the overall difference-in-differences across all initial-MSP observations was −36.7 percentage points (95% CI −38.7 to −34.4), remained between −33 and −39 percentage points in every geometry tertile, and the shuffle null spanned −3.5 to 0.0 percentage points (95% range; P = 1 × 10^−4^).

In the Figure 2f,g example, the 14-bp call removed after excluding island CpGs had been supported by four protected observations, only two of which were non-CpG.

### FiberBrowser interface

The FiberBrowser interface is a deployable application for inspecting and comparing single-molecule datasets. Indexed retrieval and display sampling limit rendering work, while sorting, filtering, clustering and co-occupancy use all eligible molecules and peak scanning uses the displayed molecules. Footprint inference is delegated to the shared FiberHMM engine, with stage progress, checkpoint reuse and report export exposed in the interface. Reversible curation, saved sessions and annotated-BAM export preserve an auditable path from the underlying observations to displayed results.

The FiberBrowser interface is implemented as a Python server with a browser-based front end and a Python client sharing one API; it is distributed with a Dockerfile and example datasets. Indexed BAM and bigBed molecule datasets can be displayed with BED, bigBed, bigWig^40^, GTF/GFF, FASTA and Hi-C reference tracks. Molecular Annotation (MA, AQ and AN), standard modification (MM and ML) and supported legacy tags are decoded into molecule objects carrying mapped blocks, informative sites, events, intervals, scores and provenance. The molecules analyzed for the specified region are held independently of the subset sampled for rendering.

The genome panel supports multiple FASTA and annotation sets with distinct contig names, and searchable BED3, BED6 and BED12 annotations, including exon blocks. Individual windows can navigate independently using local gene or coordinate search. Clustering selected windows fits one shared model to common window-relative accessibility features, retaining assignments and source identities across chromosomes or haplotypes. Users supply comparable windows; the procedure does not infer coordinate correspondence or perform liftover.

Population operations run on the server-side working set: molecules are clustered by accessibility pattern, summarized as cluster metaprofiles and co-accessibility maps, and compared across datasets with source identity retained. Sequence and motif views, coverage-based autozoom and primer design support locus inspection. In the footprint panel, chemistry and platform are read from the established FIBERHMM-CHEMISTRY:v1: BAM header comments, with supported legacy metadata also recognized. Missing metadata disables inference and links to Dataset info, where the user can declare it and optionally persist canonical comments to the BAM. Conflicting metadata cannot be silently overridden. A compatible FiberHMM installation is required.

At individual regulatory elements, footprint inspection links interval size, position and evidence to the underlying sequence; motif overlays and sequence export help interpret a footprint after it has been detected, without requiring a factor identity to define the original call. User-defined interval labels and selected molecule groups carry into profiles, clustering and co-occurrence analyses, and every operation leaves the source data unchanged.

### Haplotype and inserted-sequence examples

Haplotype examples used haplotagged (HP) PacBio Fiber-seq alignments re-called with FiberHMM 3.0. FiberBrowser joint k-means clustering (K = 2) used accessibility over the *SNRPN* differentially methylated region core (chr15:24,823,417–24,824,334) or the *RPLP0* promoter core in both haplotype panels; per-haplotype cluster counts were compared by two-sided Fisher’s exact test. CpG methylation used the PacBio 5mC calls in the input BAM, with a CpG counted as methylated at ML ≥ 128. Allelic footprints were screened at phased heterozygous SNVs within JASPAR 2024 motif cores. A molecule spanning the motif was counted as bound when a single FiberHMM footprint covered the complete motif core, and haplotypes were compared by two-sided Fisher’s exact test with Benjamini–Hochberg correction across all 27,231 tests. Replication used the same motif coordinates in other libraries heterozygous for the variant. For display, molecules were split by the base at the variant with the FiberBrowser variant scan (minor-allele fraction at least 0.1, depth at least 20, both strands). Insertions of at least 100 bp supported by at least eight molecules (MAPQ ≥ 30) were identified from CIGAR operations, and the inserted sequence was taken as the consensus of carrier molecules, which FiberBrowser expanded as a separate allele for display. Insert accessibility was measured from FiberHMM MSPs in insert coordinates, and a nucleosome-free region was counted on a molecule when an MSP of at least 150 bp covered it. The footprint sequence was scanned with JASPAR 2024 CORE vertebrate matrices^41^ (pseudo-count 0.8; background A and T 0.3, C and G 0.2), with P values from the exact score distribution.

### Promoter CNN cell-identity transfer

A five-class promoter classifier was trained on Hia5 Fiber-seq from GM12878 (LCL), H1, H9, K562 and HepG2 and applied to 12 individual HG002 scDAF-seq cells^15,42^. Validation used independent H1, H9 and K562 libraries and parent-molecule-disjoint 80:20 training–validation splits for GM12878 and HepG2. scDAF-seq molecules were reconstructed as physical duplexes and processed with the nucleosome and footprint callers described above.

Single-molecule inputs were 5-kb hg38 promoter windows oriented by transcription-start-site strand; the training panel contained 101,318 supported windows. Each observation was required to span both window ends, cover at least 4,950 bases and contain no unobserved gap longer than 30 bp. At 1-bp resolution, the input encoded nucleosome and subnucleosomal protection, soft encodings of nucleosome-free gap length, reference A/C/G/T sequence gated by footprints or regulatory gaps, and observation validity. The same reference sequence was used for both assays.

The residual CNN^43,44^ used 32 channels and nine kernel-9 convolutional blocks with dilations 1, 2, 4, 8, 16, 32, 64, 64 and 128, giving a receptive field spanning the 5-kb input. Mean and maximum features across 25 positional bins, together with feature fractions, were projected to a 32-dimensional local representation, and learned locus-specific weights related these representations to the five reference classes. Contributions were averaged within promoters, within overlapping promoter groups and across observed groups to obtain one prediction per cell.

Synthetic reference cells were assembled by sampling one or two independent molecules per promoter, with promoter coverage varied during training. Training minimized class-balanced cross-entropy with AdamW (learning rate 5 × 10−4; weight decay 0.01). Checkpoints were selected solely by mean cross-entropy across ten fixed Hia5 validation profiles, comprising one- and two-molecule profiles for each reference class; scDAF-seq predictions did not enter fitting or model selection. The selected footprint model was obtained after 1,540 optimizer updates. Its expanded reference evaluation used 160 fixed pseudo-cells: four deterministic draws for each class, one- or two-molecule sampling and promoter retention of 8%, 25%, 50% or 100%. Each scDAF-seq cell was classified separately from its eligible duplex observations without fitting any classifier parameter to scDAF-seq. Class scores are softmax-transformed logits, and the LCL margin is the LCL logit minus the largest competing-class logit.

### Raw-observation comparisons

The matched raw-input CNN replaced FiberHMM interval channels with binary modification events, assay-specific observable-substrate masks, reference A/C/G/T sequence and observation validity. Raw events remained at their original genomic positions without smoothing, rate normalization, quality-score channels or inferred intervals. Architecture, promoter-spanning molecules, reference splits, synthetic-cell sampling, batch size and initial optimizer settings matched the footprint CNN. The raw checkpoint was selected solely on held-out Hia5 reference data at update 3,780; training stopped at update 3,798 after reference accuracy saturated. Cumulative logged training and loading time to the selected checkpoint was 157.4 h for raw input and 45.9 h for footprint input. This comparison measures time to the selected checkpoints, rather than a minimum time to a common convergence threshold. scDAF-seq was not used for training or checkpoint selection. Training histories for both CNNs are shown in Supplementary Fig. 8g.

### Molecular sampling requirements for cell identification

Figure 5b compares the two CNNs at 100% promoter retention using 40 matched pseudo-cells (four draws for each of five classes at one- and two-molecule sampling). The fixed reference evaluation also included 8%, 25% and 50% retention, giving 160 held-out pseudo-cells: the footprint CNN classified 151 (94.4%) correctly, with all nine errors at 8% retention and all 120 pseudo-cells at 25–100% retention correct, and the raw-observation CNN classified 158 (98.8%) correctly.

To measure prediction at sparse molecular depth, the fitted footprint CNN was held fixed and applied to 20 deterministic nested molecule subsets of each of the 12 HG002 scDAF-seq cells at 0.01%, 0.02%, 0.05%, 0.1%, 0.2%, 0.5%, 1%, 2%, 5%, 10%, 25%, 50% and 100% of eligible promoter-spanning molecules, retaining all promoter windows of a physical duplex together and aggregating contributions as for complete cells (240 predictions per depth). The footprint CNN assigned all 12 complete cells to LCL; at 5% and 1% of molecules, 236 and 201 of 240 predictions were lymphoblastoid, and at 0.01%, with one or two retained molecules per cell, 58 of 240 (Fig. 5c). The raw-observation CNN, evaluated on matched selections at 1–100% input, assigned 22.5%, 5.4% and 0% of predictions to LCL at 1%, 25% and full input, compared with 99.6% and 100% for the footprint CNN at 25% and full input. Bands in Figure 5c show the 2.5th–97.5th percentiles across draws. Model architecture, training and checkpoint selection are described above and in Supplementary Fig. 8a–c,g.

### Masked-state representation transfer

A 343,203-parameter, three-layer masked-token transformer^45^ was trained on FiberHMM intervals from the HG002 PS00166 Hia5 Fiber-seq dataset. Autosomal maternal and paternal contigs were divided into 500-kb blocks, with 10, 2 and 4 fixed slots per haplotig assigned to training, validation and testing. Each example contained exactly 32 nucleosome, accessible (MSP) or subnucleosomal-footprint interval tokens, with 40% of token types masked in contiguous spans of three to five tokens. The model used a 96-dimensional embedding, four attention heads, a 384-dimensional feed-forward layer, sinusoidal base-pair positions and a five-token convolutional kernel. Training used AdamW for 12,000 steps with batch size 384, peak learning rate 3 × 10^−4^, 500 warm-up steps and cosine decay. The checkpoint was selected exclusively on Hia5 validation blocks. Tokenization used the called intervals directly.

The frozen model was evaluated on the Hia5 test blocks and on 254,540 windows from all 12 HG002 scDAF-seq libraries restricted to the same predeclared test blocks; no scDAF-seq data entered model fitting, token quantization or checkpoint selection. Comparators were a first-order Markov model supplied with the masked-span hint, span-only and Markov-only models, the Hia5 class marginal, the trained network after shuffling visible token types within each fiber, and an untrained network. Balanced accuracy and class-specific recall were calculated across masked token types. Confidence intervals and the paired scDAF-seq-minus-Hia5 difference were obtained by resampling 500-kb blocks; 172 blocks contained test windows from both assays.

For the masked-token transformer (Supplementary Fig. 8d–f,h), the prediction targets are the footprint caller’s interval labels; span-only and order-shuffled controls (Supplementary Fig. 8e) evaluate how much of its accuracy comes from token lengths and ordering.

### Model training records and supplementary diagnostics

The promoter CNN architecture, classwise reference confusion and optimization history are shown in Supplementary Fig. 8a–c. The confusion matrix uses the 160 fixed reference pseudo-cells (32 per class). The history distinguishes individual training losses, their 28-update rolling mean and selection cross-entropy on ten fixed Hia5 profiles. The selected update was 1,540 and training stopped at update 1,680. Selection loss and expanded 160-profile evaluation loss describe different cohorts.

The transformer architecture, sequence-order and baseline comparisons, and per-library transfer are shown in Supplementary Fig. 8d–f. Training used weight decay 0.01, with validation every 500 updates (minimum validation loss 1.190). The twelve-library evaluation uses one fixed checkpoint, whose identity and configuration accompany the figure source.

### Statistics and reproducibility

Resampling and validation respected the measurement unit. Controlled-recovery intervals resampled genomic context blocks; SsDddA emission models left out entire scDAF-seq libraries, with covariance or bootstrap resampling grouped by physical duplex or library as specified for each analysis. CTCF ChIP comparisons used site-level scores with 2,000 whole-genomic-block bootstrap samples for uncertainty and paired AUROC differences. Matched-assay profile and molecule comparisons resampled equal numbers of molecules within locus and assay. Source tables retain the prespecified metrics and all effect directions, and commands, seeds, input and model hashes, cohort definitions and exclusions are recorded in machine-readable manifests. Software tests covering saved numerical replays, MA/AQ/AN BAM round trips, reverse-orientation modification handling, masked duplex recall, installed-wheel execution and browser workflows are included with the software snapshot.

## Supplementary Material

Supplement 1

## Figures and Tables

**Figure 1 |:**
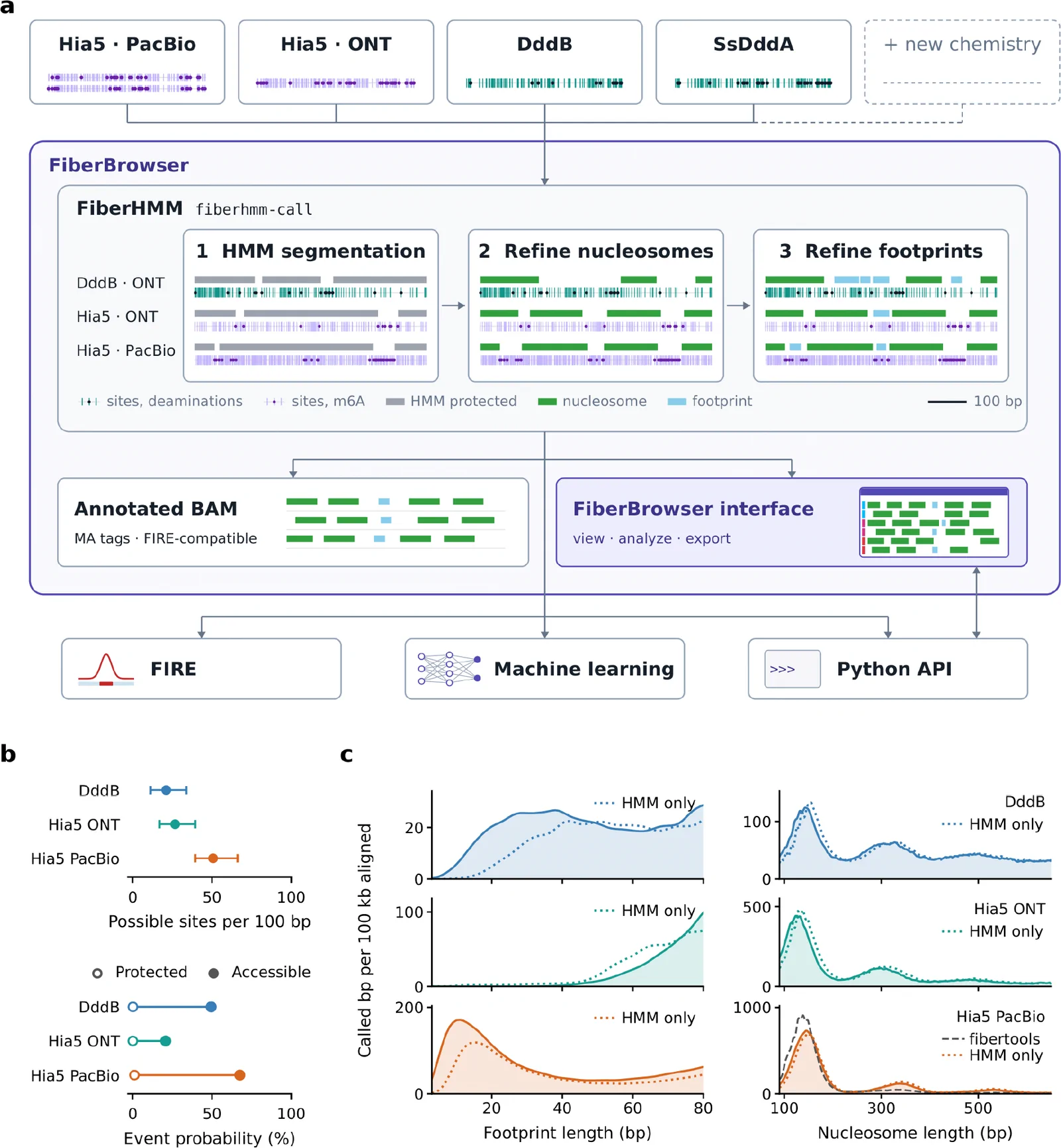
FiberHMM calls nucleosomes and footprints across single-molecule chromatin assays a, The FiberBrowser framework. Reads from each supported chemistry (Hia5 PacBio, Hia5 ONT, DddB and SsDddA; new chemistries are added with a new preset) are processed by FiberHMM (fiberhmm-call) in three steps, shown on one real 600-bp window per assay (DddB ONT at *hb* and Hia5 ONT in *Drosophila* embryos; Hia5 PacBio in K562; scale bar, 100 bp): HMM segmentation of each molecule into protected (gray) and accessible DNA, nucleosome refinement at linkers (green) and footprint refinement at LLR 5 (sky blue). Ticks, enzyme informative sites; black dots, deaminations; purple dots, m6A. Calls are written to the input BAM as Molecular Annotation (MA) tags alongside the raw observations. They are read by the FiberBrowser interface (Fig. 3), which runs the same engine and is driven by the Python API, and by downstream analyses including FIRE and machine learning (Fig. 5). b, Median possible sites per 100 bp (10th–90th percentiles) and mean event probabilities in protected and accessible DNA across the informative sites of the plotted molecules. c, Call-length distributions at LLR 5 for footprint-scale (3–80 bp) and nucleosome-scale (90–650 bp) intervals, as called bp per 100 kb aligned per 1-bp bin. DddB, 86,591 molecules from 22 *hb*, *ind*, *scyl* and *chrb* amplicon libraries; Hia5 ONT, 11,162 *Drosophila* yw embryo molecules (2–4 h); Hia5 PacBio, 3,000 K562 molecules. Dotted curves show HMM-only calls on the same DddB, Hia5 ONT and Hia5 PacBio molecules; dashed gray, fibertools ft add-nucleosomes on the matched PacBio molecules. SsDddA, whose nucleosome calls require the rotational refinement and duplex calibration introduced in Fig. 2, is characterized in Fig. 2 and Supplementary Fig. 1b.

**Figure 2 |:**
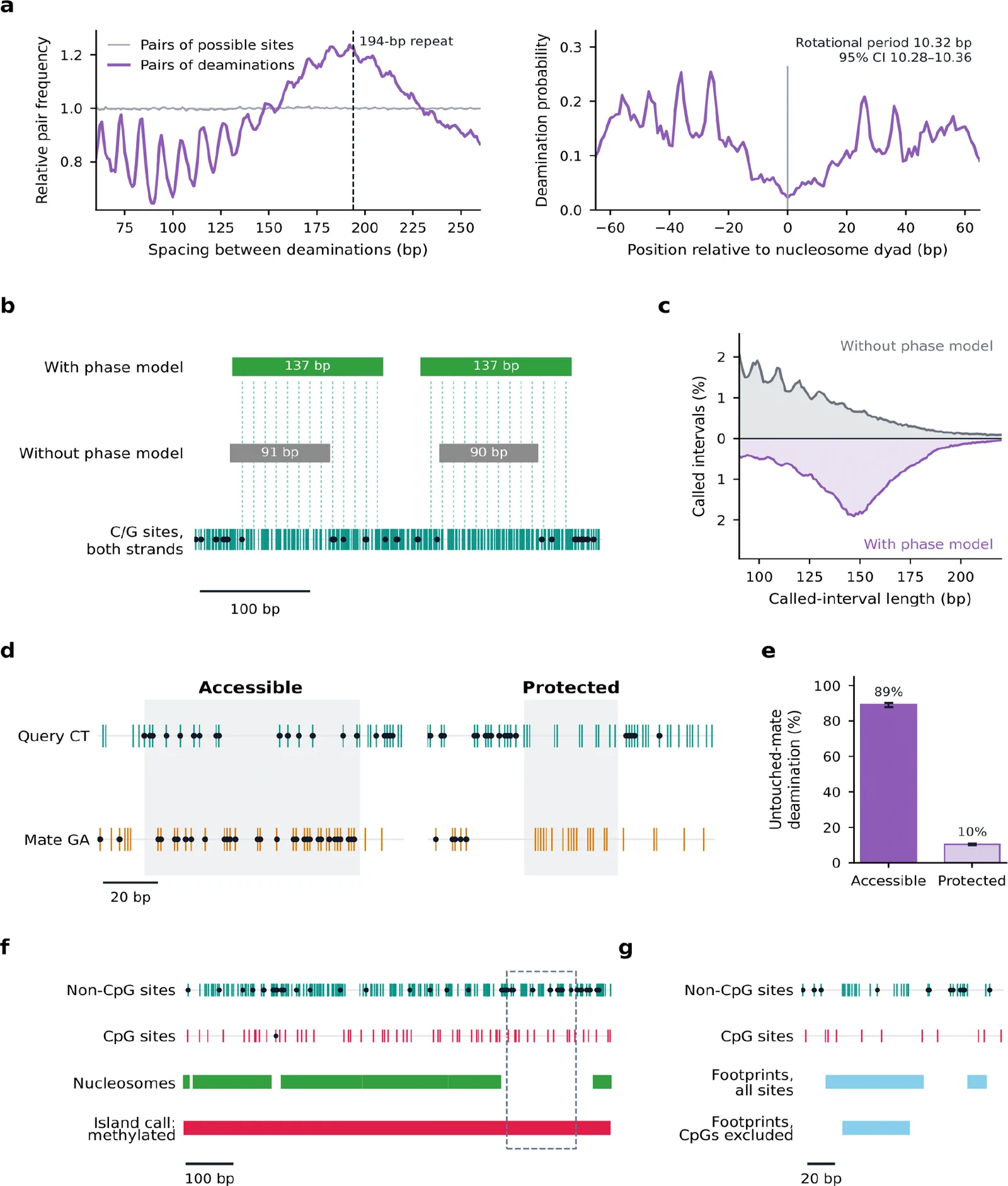
SsDddA rotational inference, duplex validation and mCG recovery a, Deamination-to-deamination spacing (left) and deamination probability relative to inferred nucleosome dyads (right), normalized to possible C/G sites. Dashed guide, 194-bp repeat; rotational period 10.32 bp (95% CI 10.28–10.36). b, One scDAF-seq molecule called without and with the phase model; C/G sites from both strands are pooled and the observations are identical. c, Called-interval length distributions for 20,915 molecules without and with the phase model (2.26 and 4.60 intervals per mapped kb, an increase that mainly reflects merged intervals of 250 bp or more, outside the plotted range, being resolved into individual particles; Supplementary Fig. 3a,b). d, Physical duplexes with an accessible and a protected query interval (shading, query interval; teal/amber ticks, CT/GA sites; black dots, deaminations). Strand pairing is described in Online Methods. e, Untouched-mate deamination for accessible and protected query sequence across 12 libraries and both transfer directions; error bars, library-cluster 95% CIs. f, Whole-island methylation inference for one HG002 molecule across an 890-bp CpG island (1/15 CpGs and 32/55 non-CpGs deaminated; methylated posterior 0.9996; matched HG002 LCL long-read methylation 86.4% across 59 sites). g, The dashed window in f, with footprints called using all sites and with CpGs excluded. Scale bars: b, f, 100 bp; d, g, 20 bp. Whole-island validation, Supplementary Fig. 5a–d.

**Figure 3 |:**
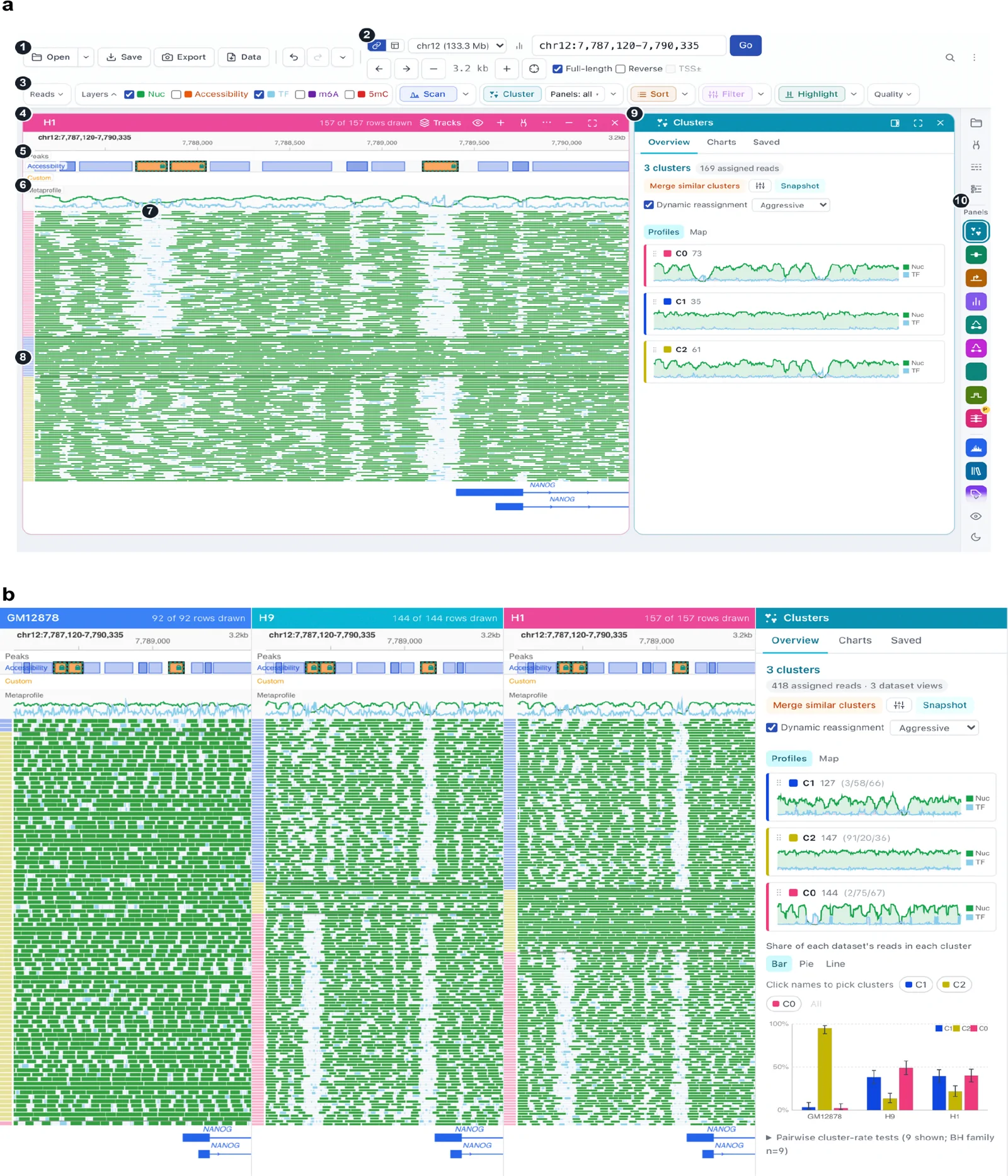
FiberBrowser turns single-molecule visualization into real-time population analysis a, The FiberBrowser interface with one H1 Fiber-seq dataset at *NANOG* (chr12:7,787,120–7,790,335). Numbered labels mark (1) file and session controls, (2) locus and panel navigation, (3) the toolbar of molecule operations (layers, scanning, clustering, sorting and filtering), (4) the dataset panel header, (5) peak and custom-feature tracks, (6) the nucleosome (green) and footprint (sky blue) metaprofile, (7) molecule rows drawn from their FiberHMM calls (green, nucleosomes; sky blue, footprints; pale, accessible DNA), (8) the cluster-membership strip, (9) the analysis sidebar, here the Clusters panel, and (10) the rail that opens analysis panels. b, Joint clustering of lymphoblastoid (GM12878) and embryonic stem cell (H9 and H1) Fiber-seq on accessibility at the *NANOG* promoter and a nearby enhancer (chr12:7,787,120–7,790,335). Left, per-dataset molecular views with shared cluster assignments (green, nucleosomes; colored strips, cluster membership) and metaprofiles. Right, the interface’s summaries of the three clusters (C0–C2), with molecule counts (GM12878/H9/H1 in parentheses), nucleosome (green) and footprint (sky blue) metaprofiles, and cluster proportions per dataset; error bars, 95% Wilson intervals with reads as units (FiberBrowser documentation).

**Figure 4 |:**
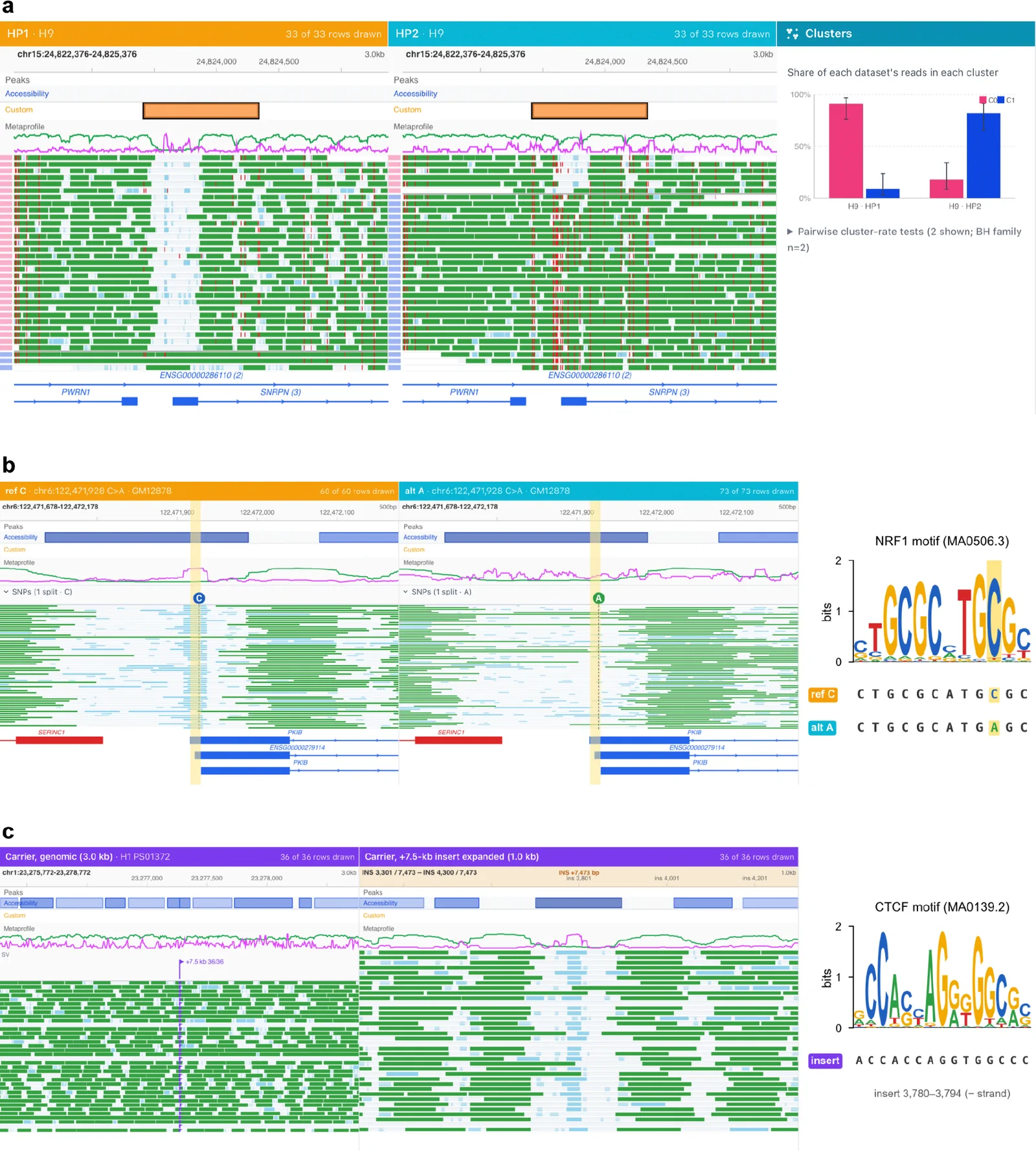
Dissecting allelic footprints, DNA methylation and non-reference structural variants on single fibers a, Parent-of-origin chromatin at the imprinted *SNRPN* locus (chr15:24,822,376–24,825,376) in H9 Fiber-seq. Phased HP1 and HP2 molecules (33 each) are shown side by side with CpG methylation (red ticks, methylated CpGs) after joint k-means clustering (K = 2) over the 917-bp differentially methylated region (orange). Right, the interface’s per-haplotype cluster proportions; error bars, 95% Wilson intervals. Accessible cluster, 30/33 HP1 versus 6/33 HP2 molecules (two-sided Fisher’s exact P = 2.2 × 10^−9^; *RPLP0*, P = 0.82). b, Allele-specific NRF1 occupancy at a heterozygous C>A variant at the bidirectional *SERINC1*–*PKIB* promoter (chr6:122,471,678–122,472,178) in GM12878 Fiber-seq. Molecules were split by the base at chr6:122,471,928 with the interface’s variant scan (reference C, 60 molecules, 59 spanning the complete motif; alternative A, 73 molecules; all drawn). The marker above each panel gives the variant base, the dashed line its position on the molecules, and yellow the NRF1 motif core. Right, the JASPAR NRF1 motif (MA0506.3) with the two alleles on the reference strand; the variant changes motif position 10 from C to A. Motif footprinted on 51/59 C and 4/73 A molecules (two-sided Fisher’s exact P = 4.0 × 10^−23^; top-ranked site among 27,231 heterozygous motif cores); replicated in both heterozygous H1 libraries (P = 8.2 × 10^−11^ and 4.1 × 10^−4^). c, A non-reference insertion at chr1:23,277,272 in H1 Fiber-seq (7,471 bp in the reference alignment; 7,473-bp carrier consensus). Left, the 36 carrier molecules at the insertion site (3 kb; flag, insertion). Right, the same molecules with the insertion consensus expanded at insert positions 3,300–4,300 (1 kb), showing a nucleosome-free region at insert positions 3,723–3,879 with a central footprint at 3,771–3,804 on about 80% of molecules. The insert consensus partly matches MER41A and contains Alu fragments. Far right, the JASPAR CTCF motif (MA0139.2) with the inserted sequence at insert positions 3,780–3,794 (minus strand), inside the central footprint (FIMO P = 3.2 × 10^−7^, q = 0.005). Subnucleosomal footprints are sky blue.

**Figure 5 |:**
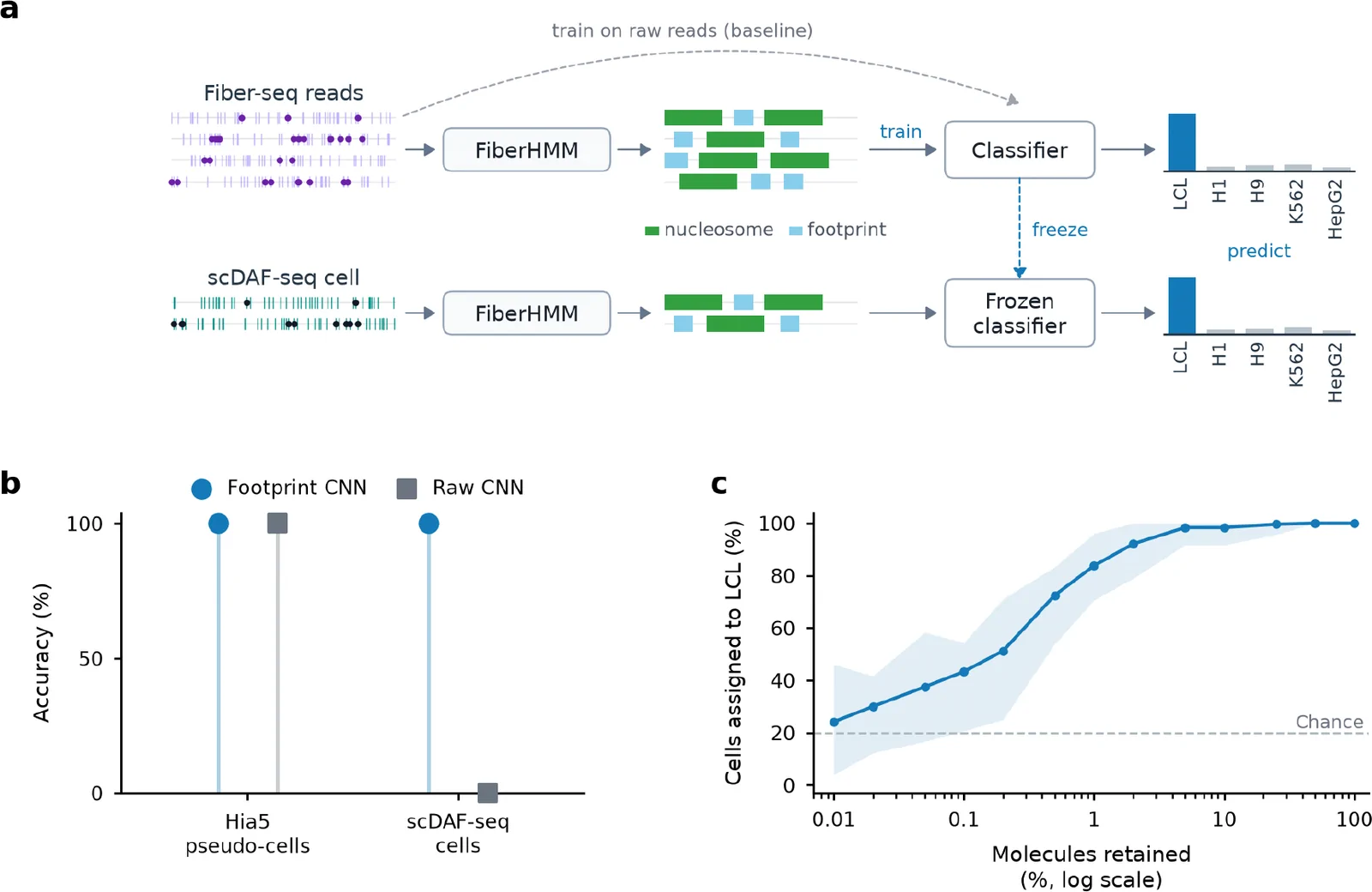
Footprint encoding supports cell identification across assays a, Schematic. Fiber-seq reads are encoded by FiberHMM as nucleosome and footprint intervals and used to train a promoter CNN that calls cell type (LCL, H1, H9, K562, HepG2); dashed arc, a CNN trained on raw reads instead. The trained classifier is frozen and applied to FiberHMM-encoded reads from single HG002 scDAF-seq cells; bar heights are illustrative. b, Full-input accuracy in Hia5 pseudo-cells (40/40 correct for both models) and scDAF-seq cells (footprint CNN, 12/12; raw CNN, 0/12). Blue, footprint CNN; gray, raw CNN. c, LCL assignment across 13 molecule-retention levels (log-scaled axis) for the footprint CNN (240 predictions per level); shading, 2.5th–97.5th percentiles across footprint-CNN draws; dashed guide, five-class chance. Training histories and the masked-token transformer, Supplementary Fig. 8a–h.

## Data Availability

Sequencing data generated for this study will be deposited in GEO before publication. These data include Fiber-seq of ENCODE cell lines, *Drosophila* DddB *hb*, *ind*, *scyl* and *chrb* amplicons, and the *Drosophila* yw 2–4-h embryo Hia5 ONT Fiber-seq dataset and its naked-DNA and untreated controls. Published scDAF-seq (BioProject PRJNA1203351; runs SRR33130336–SRR33130342, SRR33130350 and SRR33130359–SRR33130362) and *NAPA*/UBA1 datasets are from Swanson et al.^15^. Previously published GM12878 Fiber-seq data are described in the cited Fiber-seq studies^19,20^. CTCF ChIP-seq peak sets were obtained from ENCODE^34^ (K562, ENCFF430KTH; GM12878, ENCFF635MMB; H1, ENCFF414GZI; H9, ENCFF152GTF; HepG2, ENCFF008COY). HG002 LCL Fiber-seq (PS00166) is publicly available as part of the HG002 FIRE dataset (s3://stergachis/public/FIRE/broadly-consented/)^20^; per-CpG methylation calls (pb-CpG-tools v3.0.0, HG002 v1.1a) were generated as in Tullius et al.^46^. Plotting tables, model configurations and analysis provenance accompany the source data.

## Code availability

FiberBrowser (interface and Python API) is released and available at https://github.com/fiberseq/FiberBrowser. Its analytical engine, FiberHMM, including the calling, pairing and CpG tools, is released and available at https://github.com/fiberseq/FiberHMM under the MIT license. The repositories provide installation instructions and workflow documentation. Software versions and model identifiers for individual analyses accompany the source data.
