# Supplementary material for: FiberBrowser: a unified framework for single-molecule genomics
Source: bioRxiv. 2026 Oct 31:2026.10.29.762797. Preprint. [Version 1] doi: 10.64898/2026.10.29.762797 (PMC13734078; doi:10.64898/2026.10.29.762797)
Supplement: Supplementary file 1 [file NIHPP2026.10.29.762797v1-supplement-1.pdf]

bioRxiv preprint doi: https://doi.org/10.64898/2026.10.29.762797; this version posted October 31, 2026. The copyright holder for this preprint (which was not certified by peer review) is the author/funder, who has granted bioRxiv a license to display the preprint in perpetuity. It is made available under a CC-BY 4.0 International license.

Supplementary Information

Supplementary Figure 1

Emission calibration and FIRE interoperability

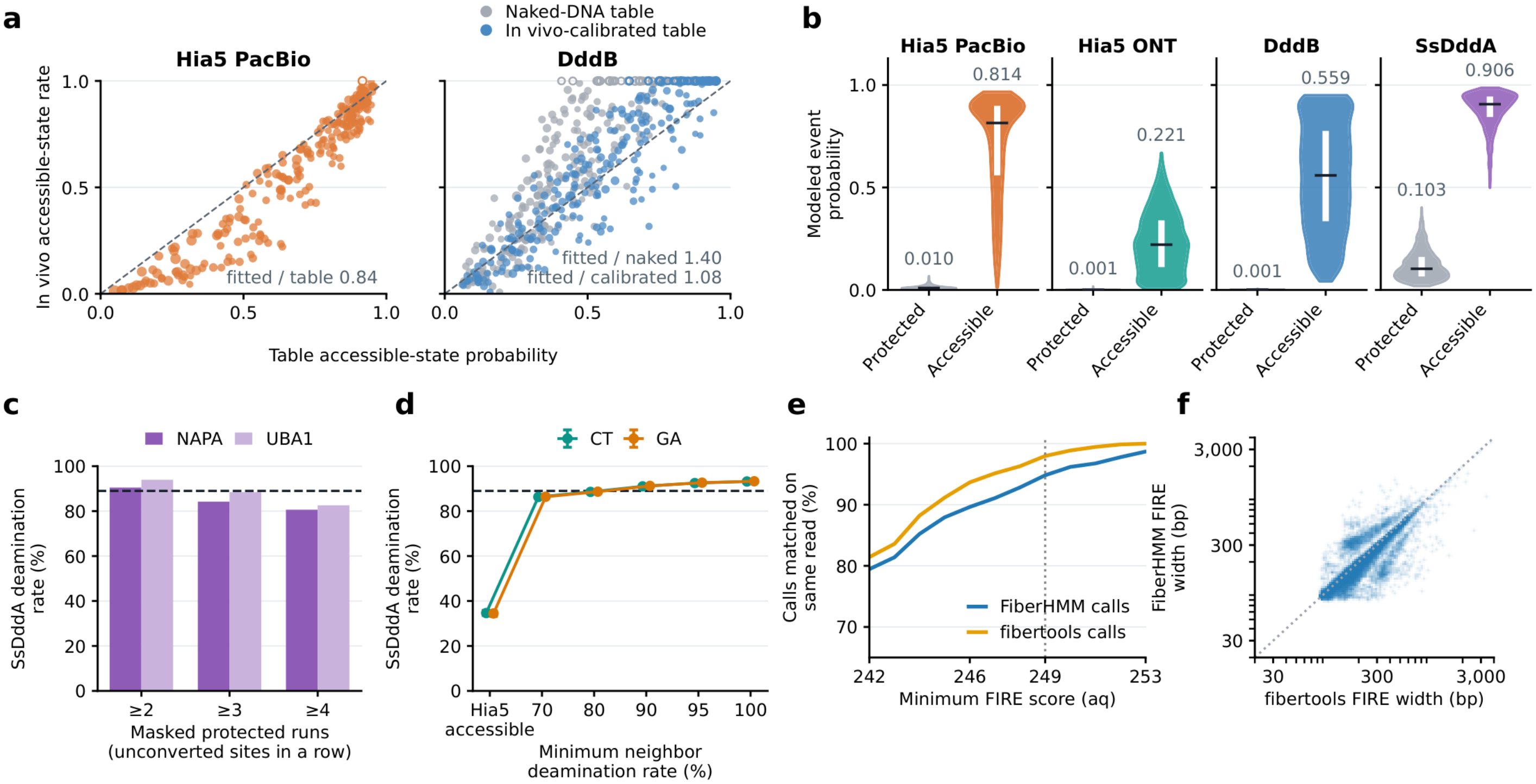

bioRxiv preprint doi: https://doi.org/10.64898/2026.10.29.762797; this version posted October 31, 2026. The copyright holder for this preprint (which was not certified by peer review) is the author/funder, who has granted bioRxiv a license to display the preprint in perpetuity. It is made available under a CC-BY 4.0 International license.

Supplementary Figure 1 | Emission calibration and FIRE interoperability

a, In vivo accessible-state calibration. In vivo accessible-state rates by five-base context against the emission tables, at the *hb*, *ind*, *chrb* and *scyl* amplicons (wild-type 2–4-h embryos; Hia5 PacBio 2–4 h). For each enzyme, the accessible-state rate per context was fitted as P(event) = $a \cdot p_{acc} + (1 - a) \cdot p_{prot}$, where a is the fraction of molecules from an independent half of the same enzyme's data whose MSP covers the site outside called footprints. Hia5 (left) reached 0.84 of its table in this per-context fit (the MSP-interior comparison in Methods gives 0.56–0.61); DddB (right) exceeded the naked-DNA table 1.40-fold (gray) and the in vivo-calibrated table 1.08-fold (blue). Relative to Hia5, this places DddB at 1.67× the naked table (95% locus bootstrap 1.58–1.75; per locus 1.45–1.74); the table level was set conservatively at 1.29×. 256 five-base contexts per enzyme; point area, observed sites; open markers, fits at the upper bound (43 DddB contexts).

b, Sequence-context emission distributions. Modeled event probabilities for all 4,096 contexts of each chemistry, not weighted by how often each context occurs; gray, protected; colored, accessible; black line, median; white bar, interquartile range.

c, Hia5-accessible-region-based SsDddA rate sensitivity. *NAPA* and *UBA1* deamination rates after excluding runs of two, three or four unconverted SsDddA sites. The dashed line marks the reported physical-duplex accessible rate of 89%.

d, Genome-wide Hia5-guided rate sensitivity. Test-chromosome CT and GA event fractions under the independent Hia5-accessible mask alone and with additional focal-site-excluded SsDddA neighbor-rate thresholds. All 6,022 genomic blocks and twelve matched scDAF-seq libraries entered the predefined splits. Error bars are 95% chromosome-group bootstrap intervals, with both haplotypes resampled together. The dashed line marks the 89% duplex reference.

e, Same-read FIRE concordance versus minimum FIRE score (aq). Each curve is the fraction of one caller's FIRE calls matched by a positive FIRE call from the other segmentation on the same read, among 9,781 common LCL (GM12878) reads. At aq ≥ 249, concordance is 98.0% for fibertools and 94.8% for FiberHMM. Final MA:msp coordinates match FIRE's as/al input exactly.

f, Width agreement of shared FIRE calls. Paired widths for 17,340 one-to-one shared calls; the diagonal marks equality. Median absolute difference is 8 bp (interquartile range 4–21 bp). Axes are logarithmic.

bioRxiv preprint doi: https://doi.org/10.64898/2026.10.29.762797; this version posted October 31, 2026. The copyright holder for this preprint (which was not certified by peer review) is the author/funder, who has granted bioRxiv a license to display the preprint in perpetuity. It is made available under a CC-BY 4.0 International license.

Supplementary Figure 2

Footprint threshold, recovery and caller benchmarks

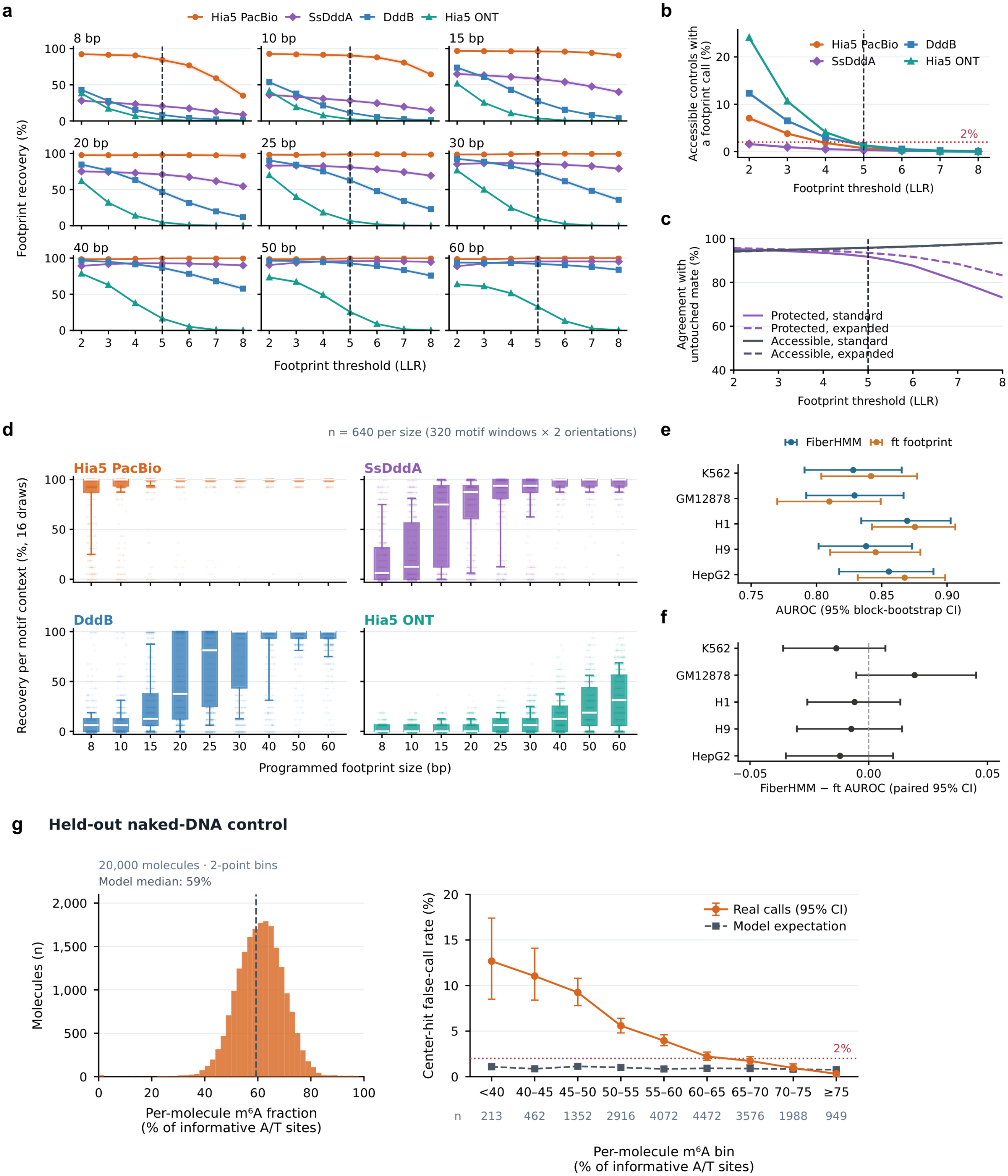

bioRxiv preprint doi: https://doi.org/10.64898/2026.10.29.762797; this version posted October 31, 2026. The copyright holder for this preprint (which was not certified by peer review) is the author/funder, who has granted bioRxiv a license to display the preprint in perpetuity. It is made available under a CC-BY 4.0 International license.

Supplementary Figure 2 | Footprint threshold, recovery and caller benchmarks

a, Protected-target LLR curves. Footprint recovery (target center covered) across LLR 2–8 for each programmed footprint size from 8 to 60 bp. Each chemistry and threshold uses 320 genomic motif contexts, two orientations and 16 draws per size. Shading gives 95% genomic-block-bootstrap intervals, and the vertical guide marks the shared LLR 5 setting.

b, Pooled accessible-control curve. Matched fully accessible windows are pooled across the nominal target-size strata because no protected target exists in these controls. The horizontal guide marks 2%; LLR 5 is the first shared integer setting below 2% for every chemistry.

c, Expanded physical-mate calibration. Default-decoder protected-window recovery and accessible-window specificity on untouched mates from two held-out libraries, comparing standard emissions (solid) with expanded exact six-base emissions (dashed); purple, protected-window recovery; gray, accessible-window specificity. The full cohort comprises 490,934 duplexes from twelve libraries with an eight/two/two training/validation/test split. Shading gives 95% physical-duplex-cluster bootstrap intervals, conditional on the two test libraries; the vertical guide marks LLR 5. Both query directions are pooled.

d, Context-level recovery distributions at LLR 5. Per-context recovery of a subnucleosomal footprint covering the programmed center, the endpoint of a, across 16 draws for all four chemistries at LLR 5 and every programmed footprint size. Each size has 640 units (320 motif windows × 2 orientations), each recalled over 16 draws.

e, AUROC for FiberHMM complete-motif calls and fibertools footprint calls (ft footprint) on 512 motifs per cell type.

f, Paired FiberHMM-minus-ft AUROC differences. Points show estimates and bars show 95% percentile confidence intervals from 2,000 paired resamples of 438 occupied 1-Mb genomic blocks. The dashed line marks zero difference. Both callers use the same eligible molecule–motif observations; all five paired intervals include zero. FiberHMM calls are scored using rule R1; the 10–89-bp complete-motif rule R3 gives identical results.

g, Held-out naked-DNA control. Left, per-molecule m6A fraction in 20,000 Hia5-treated naked-DNA molecules not used for emission fitting; dashed line, median of the model expectation. Right, fraction of fixed 800-bp windows with a footprint covering the center at LLR 5, by per-molecule m6A fraction, for real calls (95% molecule-bootstrap intervals) and for observations drawn from the accessible-state emissions at the same sites; n, molecules per bin. Overall rates are 3.6% and 0.9%.

bioRxiv preprint doi: https://doi.org/10.64898/2026.10.29.762797; this version posted October 31, 2026. The copyright holder for this preprint (which was not certified by peer review) is the author/funder, who has granted bioRxiv a license to display the preprint in perpetuity. It is made available under a CC-BY 4.0 International license.

Supplementary Figure 3

SsDddA nucleosome inference and adjacent-target dependence

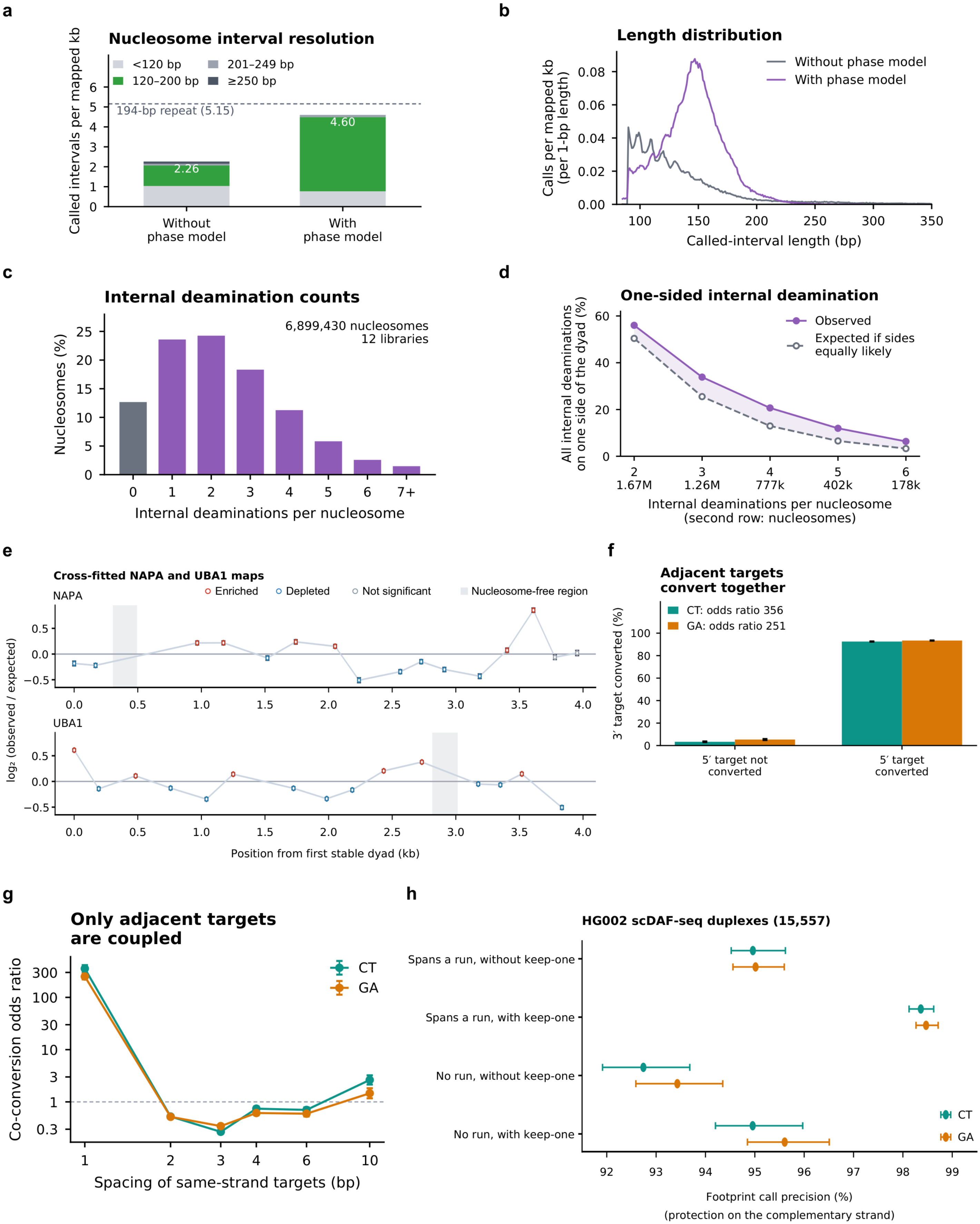

bioRxiv preprint doi: https://doi.org/10.64898/2026.10.29.762797; this version posted October 31, 2026. The copyright holder for this preprint (which was not certified by peer review) is the author/funder, who has granted bioRxiv a license to display the preprint in perpetuity. It is made available under a CC-BY 4.0 International license.

Supplementary Figure 3 | SsDddA nucleosome inference and adjacent-target dependence

a, Nucleosome interval resolution. Called intervals per mapped kb without or with the phase model, separated by span. The dashed line is 1,000/194 = 5.15 calls per kb, a spacing reference for a 194-bp repeat.

b, Length distribution with the phase model. Called-interval length distributions from the same molecules without or with the phase model.

c, Internal event-count distribution. Internal deamination counts among all 6,899,430 eligible nucleosomes from 12 libraries, including 874,439 with no internal event (12.7%).

d, Internal-event sidedness. Nucleosomes with all internal deaminations on one side of the called dyad, compared with the expectation if both sides are equally likely, for two to six events; the second row of x labels gives the number of nucleosomes in each group.

e, Cross-fitted *NAPA* and *UBA1* maps. Caller-free, cross-fitted fixed-window event burden at recurrent nucleosome sites, shown as log2(observed/expected). Error bars are the molecule-bootstrap 95% intervals. Red and blue indicate significant enrichment and depletion, respectively, at family-wise corrected $P < 0.05$; gray indicates nonsignificant positions. Gray bands mark the annotated nucleosome-free regions (NFRs) at *NAPA* chr19:47,515,050–47,515,240 and *UBA1* chrX:47,193,580–47,193,780. Positions are measured from the first stable dyad in each amplicon.

f, Adjacent targets convert together. Conversion of the 3′ target of isolated adjacent same-strand pairs (CC on CT reads, GG on GA reads), conditional on conversion of the 5′ target in the deaminated strand's orientation, at confidently accessible sites of *NAPA* SsDddA molecules (inside an MSP and at least 10 bp from any footprint or nucleosome call; unthinned informative-site sets). Odds ratios 356 (CT; 42,871 pairs) and 251 (GA; 40,028 pairs).

g, Only adjacent targets are coupled. Co-conversion odds ratio against the spacing of consecutive same-strand targets with no target between them. Shared local chromatin state would couple nearby targets at every short spacing; instead only adjacent targets are coupled, and pairs 2–6 bp apart are slightly anti-correlated.

h, Physical-duplex precision. Precision (%) of native SsDddA footprint calls in 15,557 sequence-assigned HG002 scDAF-seq physical duplexes, read as protection on the complementary strand of the same molecule, for calls that do or do not span same-strand runs, without and with keep-one. CpGs excluded; library-bootstrap 95% intervals.

bioRxiv preprint doi: https://doi.org/10.64898/2026.10.29.762797; this version posted October 31, 2026. The copyright holder for this preprint (which was not certified by peer review) is the author/funder, who has granted bioRxiv a license to display the preprint in perpetuity. It is made available under a CC-BY 4.0 International license.

Supplementary Figure 4

Physical-duplex pairing and resolution

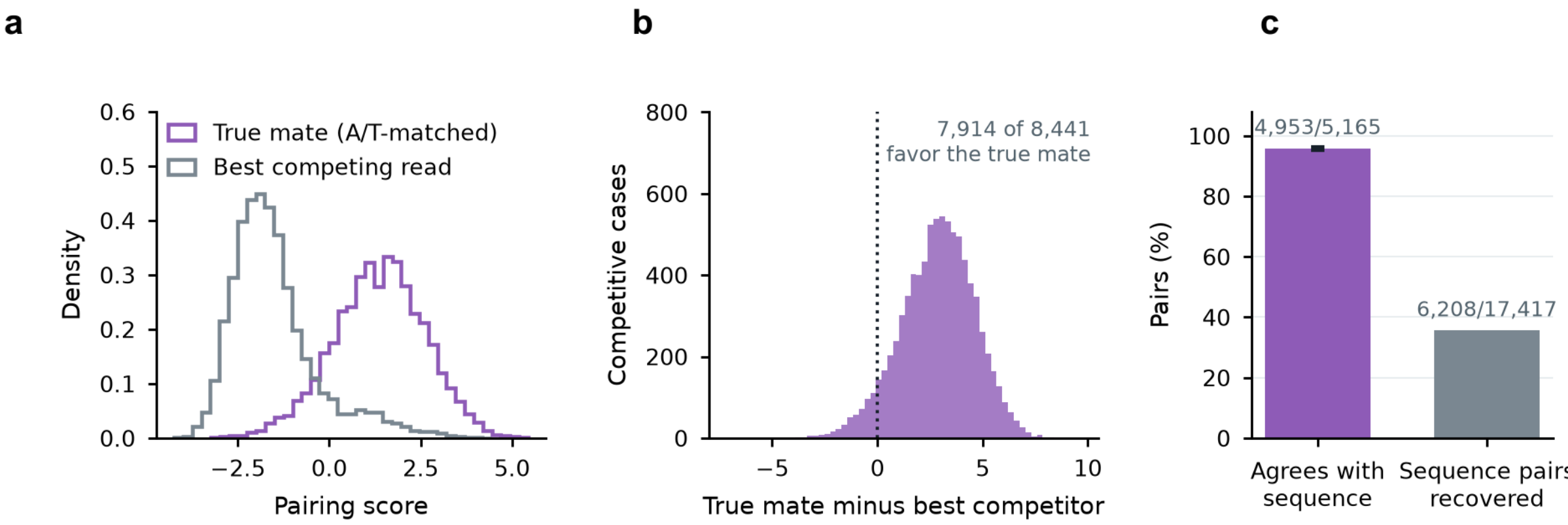

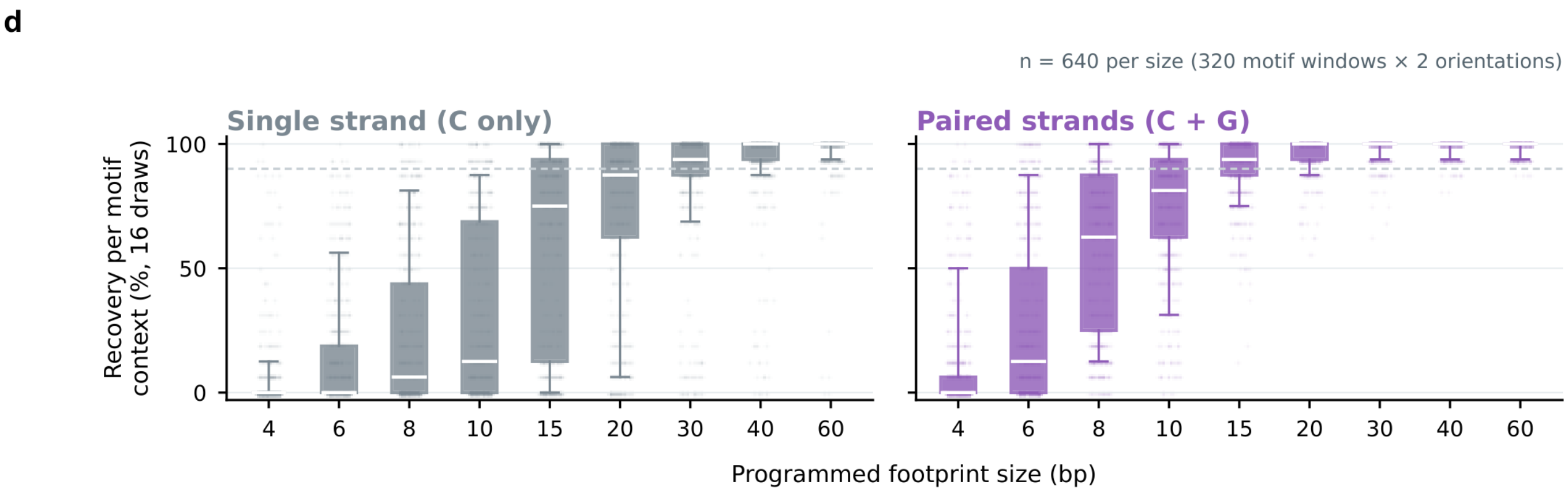

bioRxiv preprint doi: https://doi.org/10.64898/2026.10.29.762797; this version posted October 31, 2026. The copyright holder for this preprint (which was not certified by peer review) is the author/funder, who has granted bioRxiv a license to display the preprint in perpetuity. It is made available under a CC-BY 4.0 International license.

Supplementary Figure 4 | Physical-duplex pairing and resolution

Validation of footprint-only pairing against independent sequence-supported mate assignments.

a, Pairing score distributions. Fixed footprint-only scores for independently A/T-selected mates and their best read-sharing competitors in 8,441 competitive cases where both reads map uniquely to the same HG002 haplotype, across the twelve-library evaluation cohort. A/T identity supplies only the evaluation label; pairing-model fitting regions are excluded.

b, Competitive score differences. Mate score minus the best competing score in the same cases; 7,914/8,441 favor the independently selected mate.

c, Pairing agreement and recovery. At loci where both reads map uniquely to one HG002 haplotype, so that sequence can distinguish the mate from the other homolog's strand, 4,953 of 5,165 sequence-assessable footprint-selected pairs agree with the independent A/T relationship (95.9%; 95% connected-component bootstrap interval, 95.3–96.4%). Recovery is 6,208/17,417 among all independently A/T-selected pairs, including footprint-unscorable pairs. The full cohort contained 461,127 footprint-selected pairs: 454,100 (98.48%) lacked a decisive A/T mate assignment, and 77.21% lacked a homolog comparison. Sequence and haplotype were withheld from footprint pairing.

d, Resolution gained from complementary strands. Context-level recovery distributions from 320 genomic motif windows in two orientations, with 16 draws per unit, using the standard SsDddA footprint emissions and default multi-interval decoder at LLR 5 with at least three informative sites. Pairing combines the complete C+G informative-site set instead of calling only the C-bearing strand. Pooled recovery increases from 23.6% to 57.0% at 8 bp, 32.5% to 75.5% at 10 bp, and 58.4% to 91.7% at 15 bp; boxes show quartiles, whiskers the 10th–90th percentiles, and points all 640 units (320 motif windows × 2 orientations), each recalled over 16 draws.

bioRxiv preprint doi: https://doi.org/10.64898/2026.10.29.762797; this version posted October 31, 2026. The copyright holder for this preprint (which was not certified by peer review) is the author/funder, who has granted bioRxiv a license to display the preprint in perpetuity. It is made available under a CC-BY 4.0 International license.

Supplementary Figure 5

Complete-CpG-island methylation inference

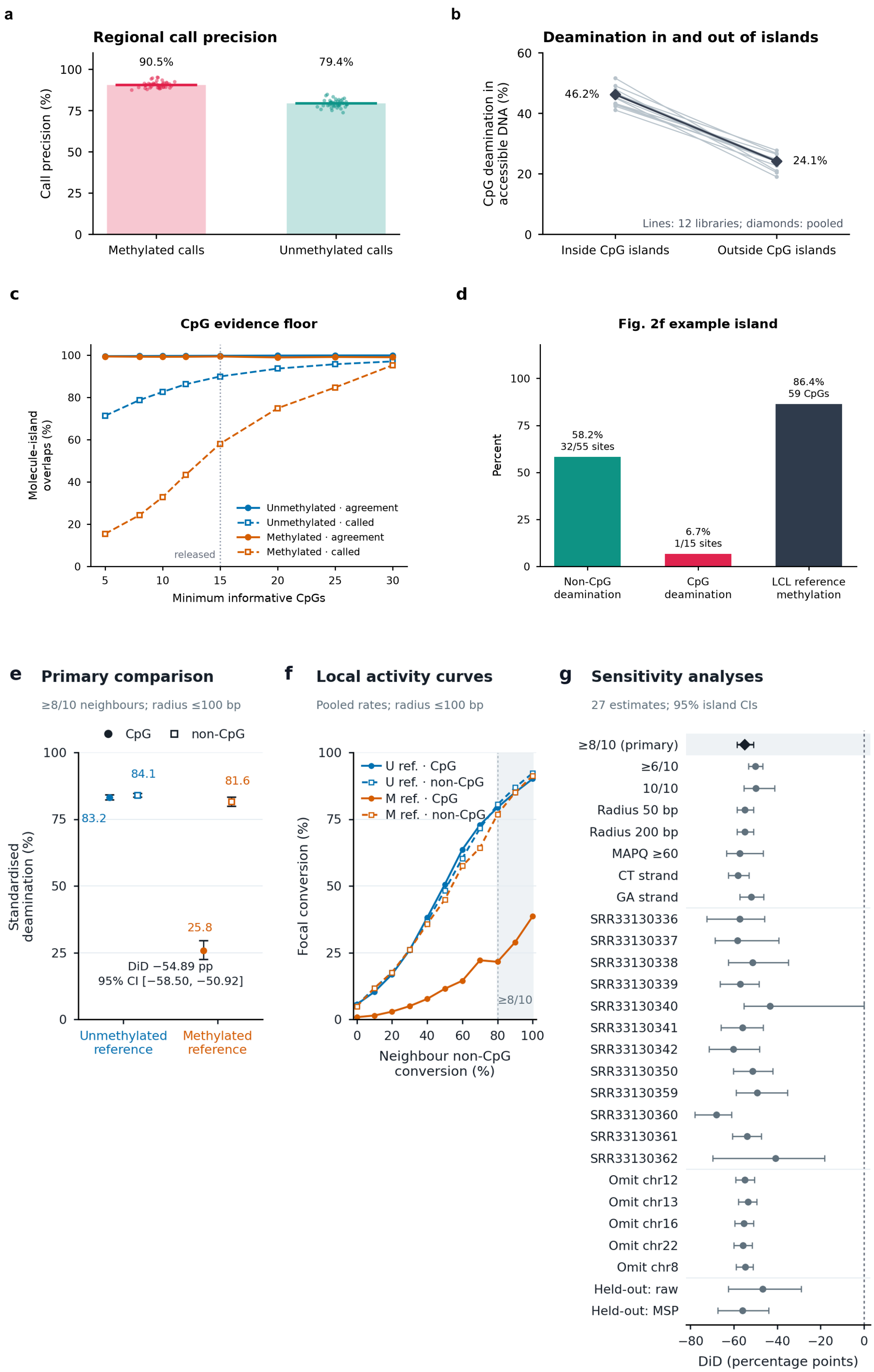

bioRxiv preprint doi: https://doi.org/10.64898/2026.10.29.762797; this version posted October 31, 2026. The copyright holder for this preprint (which was not certified by peer review) is the author/funder, who has granted bioRxiv a license to display the preprint in perpetuity. It is made available under a CC-BY 4.0 International license.

Supplementary Figure 5 | Complete-CpG-island methylation inference

a, Regional call precision. Methylated- and unmethylated-call precision in the 1-kb regional validation against the HG002 LCL reference, shown on a full 0–100% scale. Points are 46 haplotype-contig estimates; bars are means weighted by the number of truth windows (90.5% and 79.4%, respectively).

b, Deamination inside and outside islands. CpG deamination in accessible DNA inside and outside inferred CpG islands across the validation libraries; gray lines, individual libraries; diamonds, pooled.

c, CpG evidence floor. Agreement with the HG002 LCL reference among confident calls (solid) and fraction of evidence-eligible molecule–island overlaps called confidently (dashed) for unmethylated- and methylated-reference islands on validation chromosomes, as the minimum number of informative CpGs varies; posterior threshold 0.99 and at least 10 non-CpG observations. The dotted line marks the released 15-CpG floor, which was not re-selected.

d, Fig. 2f example island. Deamination of non-CpG (32/55) and CpG (1/15) sites in the Fig. 2f island and its matched HG002 LCL long-read methylation (86.4% across 59 CpGs).

e, CpG-specific loss at matched local accessibility. Standardized CpG and non-CpG deamination in methylated- and unmethylated-reference islands for focal sites with at least eight of their ten nearest non-CpG neighbors within 100 bp converted. Difference-in-differences −54.89 percentage points (95% island-bootstrap interval −58.50 to −50.92); 157,048 matched site observations across 1,051 islands and 12 libraries.

f, Local activity curves. Focal conversion against neighbor non-CpG conversion. Non-CpG curves overlap between reference states at every accessibility level, whereas CpG conversion is reduced only in methylated-reference islands. Shaded region, primary ≥8/10 tier; U, unmethylated reference; M, methylated reference.

g, Sensitivity analyses. Difference-in-differences with 95% island-bootstrap intervals for alternative neighbor requirements, radii and mapping quality, each strand, each library, each omitted test chromosome and two held-out analyses in which island state was assigned from 80% of CpGs and conversion measured at the withheld 20%, using either initial MSPs or raw non-CpG neighbors alone.

bioRxiv preprint doi: https://doi.org/10.64898/2026.10.29.762797; this version posted October 31, 2026. The copyright holder for this preprint (which was not certified by peer review) is the author/funder, who has granted bioRxiv a license to display the preprint in perpetuity. It is made available under a CC-BY 4.0 International license.

Supplementary Figure 6

FiberBrowser data library and primer design

a

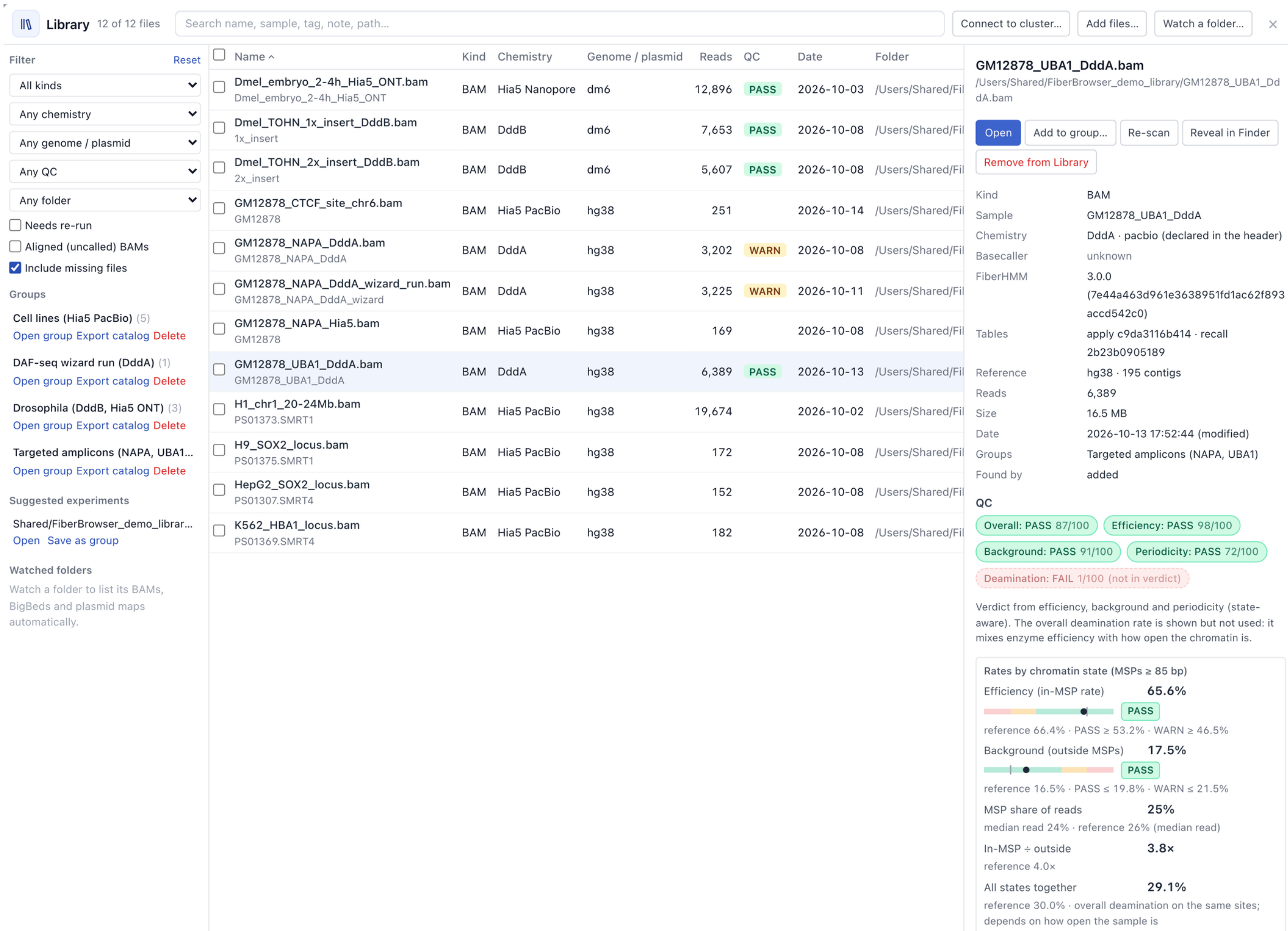

b

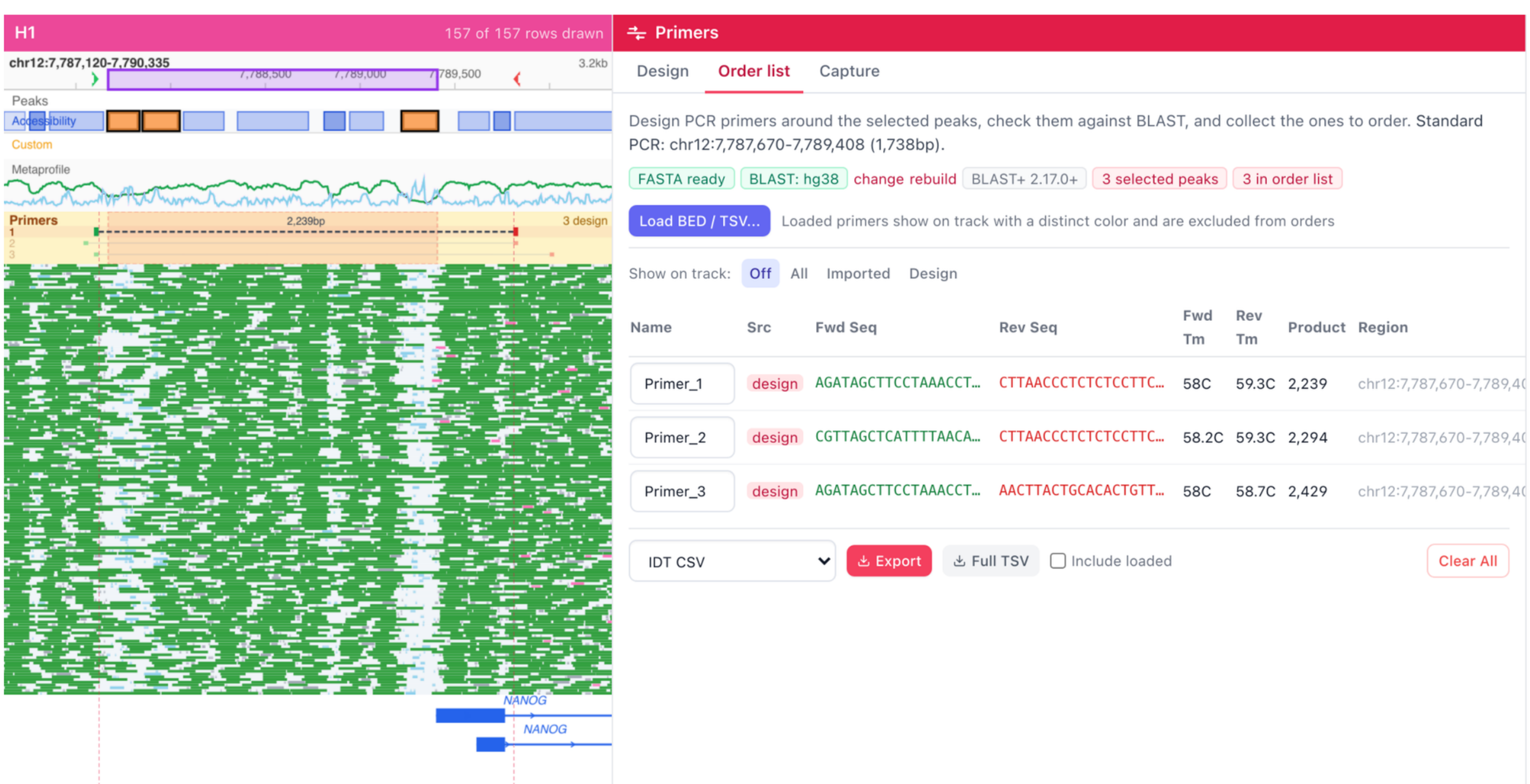

bioRxiv preprint doi: https://doi.org/10.64898/2026.10.29.762797; this version posted October 31, 2026. The copyright holder for this preprint (which was not certified by peer review) is the author/funder, who has granted bioRxiv a license to display the preprint in perpetuity. It is made available under a CC-BY 4.0 International license.

Supplementary Figure 6 | FiberBrowser data library and primer design

a, Data library. Twelve datasets from this study (Hia5 PacBio, Hia5 ONT, DddB and SsDddA, labeled DddA in the interface) organized into user-defined groups. Chemistry, genome and read counts are read from the BAM headers, and quality control (QC) compares each dataset with a chemistry-matched control; QC is shown only where such a control exists. The selected SsDddA amplicon dataset passes the efficiency, background and periodicity checks.

b, Primer design at *NANOG* in H1. Candidate amplicons are displayed above the molecular view, alongside primer sequences, melting temperatures and product lengths in the exportable order list. The displayed primers have not been experimentally validated.

bioRxiv preprint doi: https://doi.org/10.64898/2026.10.29.762797; this version posted October 31, 2026. The copyright holder for this preprint (which was not certified by peer review) is the author/funder, who has granted bioRxiv a license to display the preprint in perpetuity. It is made available under a CC-BY 4.0 International license.

Supplementary Figure 7

FiberBrowser display and analysis performance

a

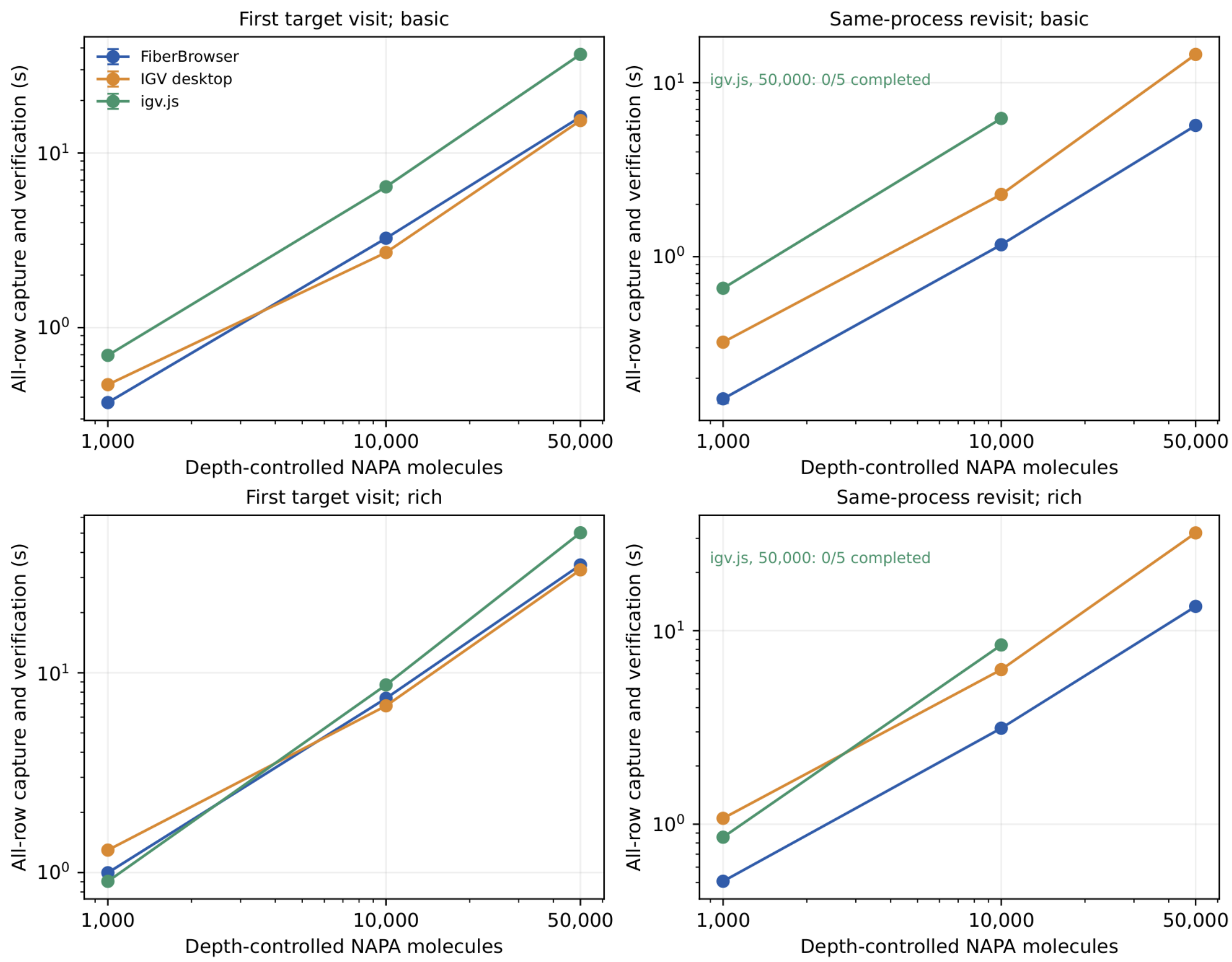

b

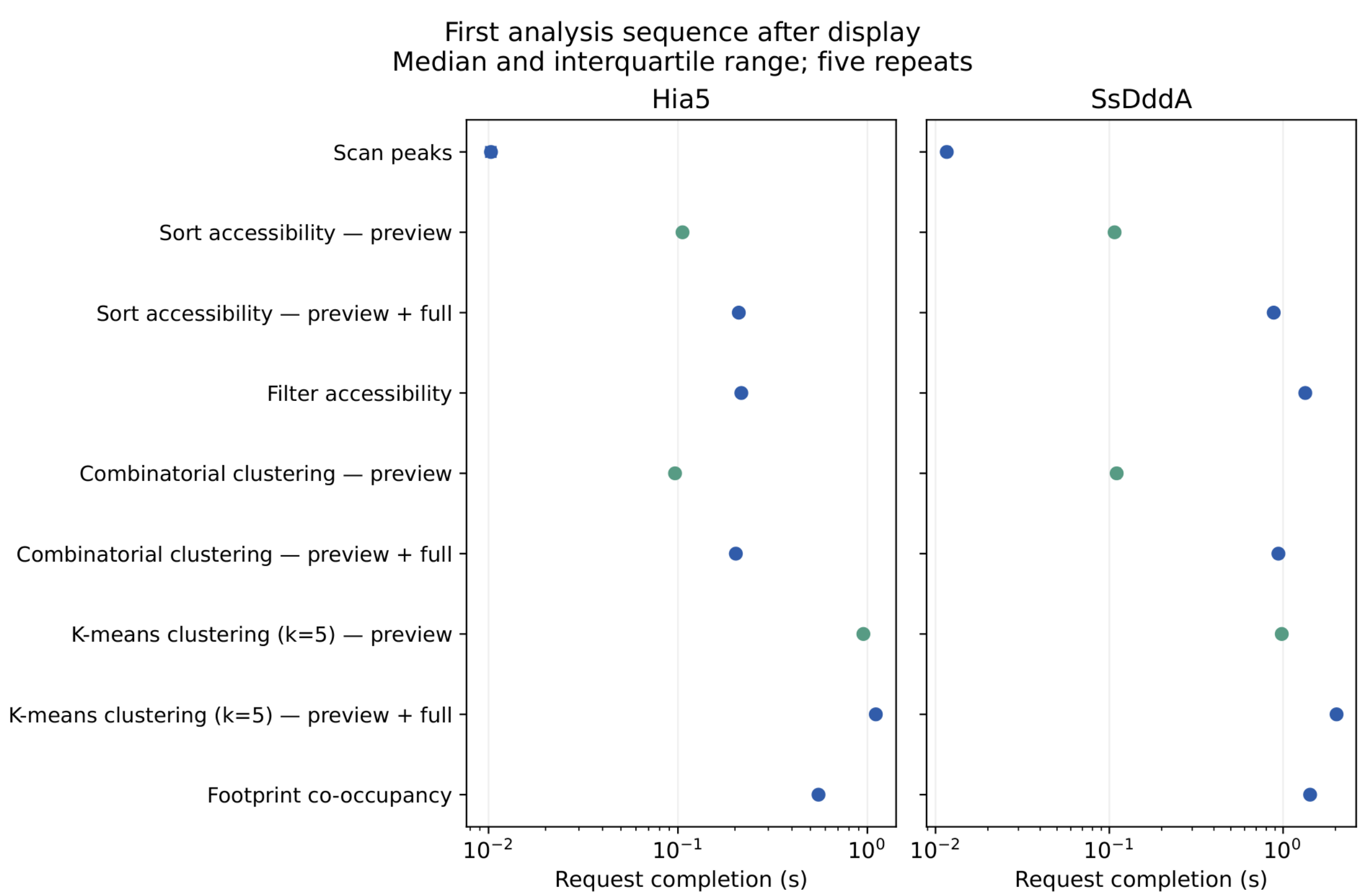

bioRxiv preprint doi: https://doi.org/10.64898/2026.10.29.762797; this version posted October 31, 2026. The copyright holder for this preprint (which was not certified by peer review) is the author/funder, who has granted bioRxiv a license to display the preprint in perpetuity. It is made available under a CC-BY 4.0 International license.

Supplementary Figure 7 | FiberBrowser display and analysis performance

a, All-read display performance. Points show the median and bars the interquartile range of five trials. The endpoint includes capture and verification of every read across bounded image tiles. First target visits and same-process revisits are shown separately. Basic mode omits additional molecular evidence; rich mode displays FiberBrowser molecular annotations or IGV mismatch evidence. These are distinct annotation workloads. Missing five-repeat groups are labeled in the plot.

b, First sequence after verified display; median and interquartile range of five trials. Preview and preview-plus-full completion are separate measurements. The repeated-sequence plot and per-stage source tables are supplied separately.

bioRxiv preprint doi: https://doi.org/10.64898/2026.10.29.762797; this version posted October 31, 2026. The copyright holder for this preprint (which was not certified by peer review) is the author/funder, who has granted bioRxiv a license to display the preprint in perpetuity. It is made available under a CC-BY 4.0 International license.

Supplementary Figure 8

Model architecture, training and frozen cross-assay evaluation

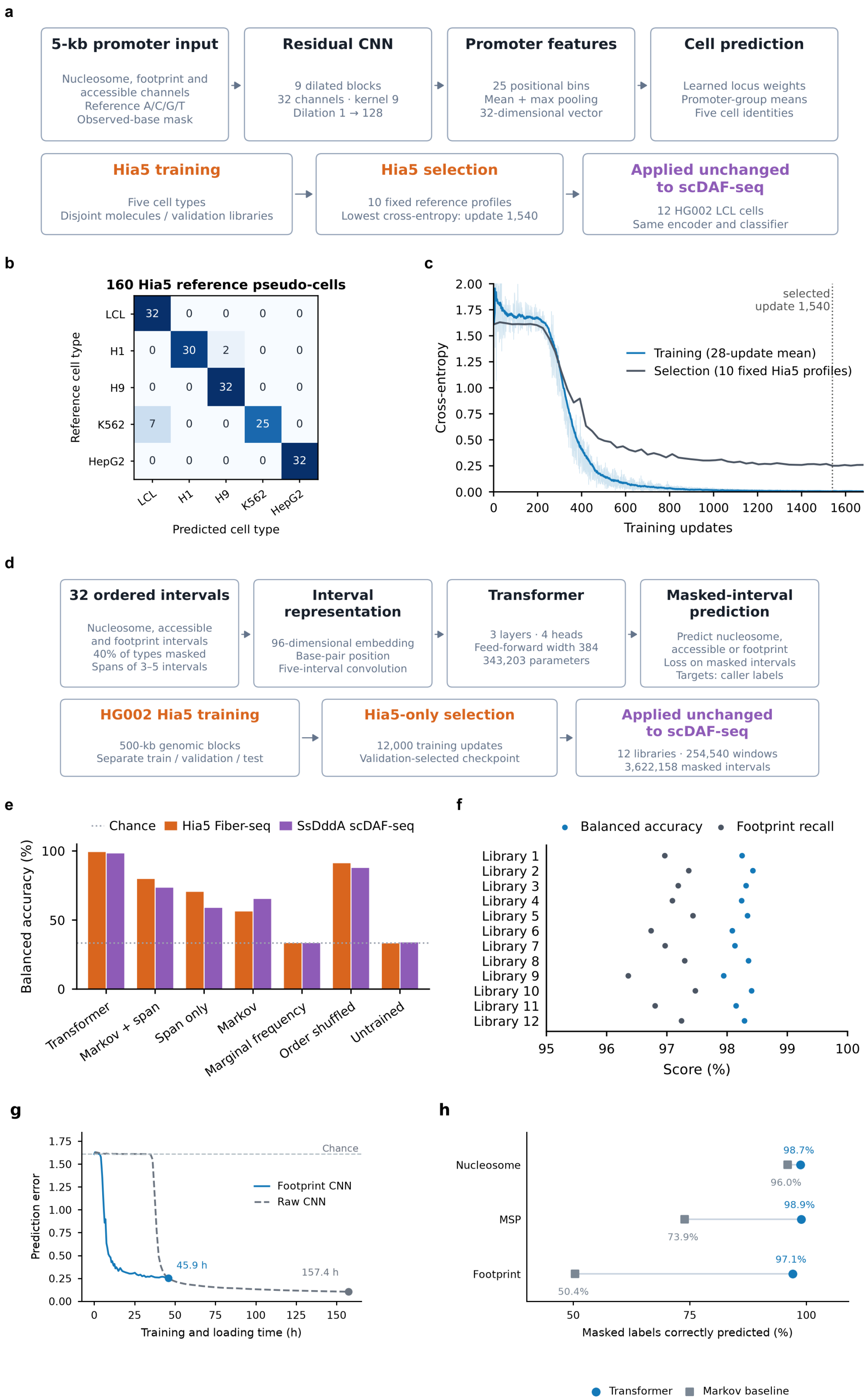

bioRxiv preprint doi: https://doi.org/10.64898/2026.10.29.762797; this version posted October 31, 2026. The copyright holder for this preprint (which was not certified by peer review) is the author/funder, who has granted bioRxiv a license to display the preprint in perpetuity. It is made available under a CC-BY 4.0 International license.

Supplementary Figure 8 | Model architecture, training and frozen cross-assay evaluation

a, Promoter CNN. Input channels (nucleosome, footprint and accessible intervals; reference sequence), dilated convolutional encoder, promoter aggregation and five-class prediction. Training and checkpoint selection use Hia5 only; HG002 scDAF-seq predictions use the frozen model.

b, Reference confusion matrix. Counts among 160 fixed Hia5 reference pseudo-cells, 32 per cell type. Rows are reference identities and columns predictions; color denotes row percentage.

c, CNN optimization and selection. Individual training cross-entropies (pale), their 28-update rolling mean (blue) and cross-entropy on ten fixed selection profiles (dark gray). The dotted line marks selected update 1,540.

d, Masked-token transformer. Ordered interval input, interval representation, three-layer attention model and masked-interval prediction. Hia5 genomic blocks are split before fitting. The frozen model is applied to twelve scDAF-seq libraries.

e, Model and order controls. Balanced accuracy for the compact transformer, Markov-plus-span, span-only, Markov, marginal-frequency, order-shuffled and untrained controls, in Hia5 Fiber-seq (orange) and SsDddA scDAF-seq (purple). The dotted line marks three-class chance.

f, Per-library transfer. Balanced accuracy and footprint recall for each of twelve scDAF-seq libraries (numbered 1–12 by SRA accession; mapping in source data), from 254,540 windows and 3,622,158 masked tokens. No scDAF-seq observations enter training or checkpoint selection.

g, CNN training histories. Prediction error (validation cross-entropy) of the footprint and raw-input CNNs against cumulative logged training and loading time. Points mark the reference-selected checkpoints (footprint, update 1,540 at 45.9 h; raw, update 3,780 at 157.4 h); dashed guide, five-class chance. Times are to the selected checkpoints, not to a common convergence threshold.

h, Masked-label transfer by interval type. Percentage of masked nucleosome, MSP and footprint labels correctly predicted in scDAF-seq by the frozen transformer and by a first-order Markov model using neighboring intervals and interval-span features (Markov baseline); 3,622,158 masked intervals across 12 libraries.

bioRxiv preprint doi: https://doi.org/10.64898/2026.10.29.762797; this version posted October 31, 2026. The copyright holder for this preprint (which was not certified by peer review) is the author/funder, who has granted bioRxiv a license to display the preprint in perpetuity. It is made available under a CC-BY 4.0 International license.

Supplementary Table 1 | Molecular Annotation encoding written by FiberHMM and read by the FiberBrowser interface

The Molecular Annotation specification developed by Mitchell R. Vollger supplies the shared interval representation. Coordinates refer to the original molecule, including reverse-aligned reads.

Tag or layer	Stored content	Interpretation
MA:Z	Original molecule length; 1-based start/length intervals; type, strand and quality declarations	Alignment supplies genomic projection.
AQ:B:C	Declared quality bytes in MA interval order	Q is a linear 0–255 byte; P is Phred-scaled. Meaning depends on the declared field.
AN:Z	Optional names aligned to MA intervals	Optional interval names; native annotations are unchanged.
nuc.Q or nuc.QQQ	Nucleosome quality; optional left/right edge qualities	Native nucleosome layer.
msp.	Accessible-patch intervals	Final coordinates are also written to as/al for fibertools/FIRE.
tf.QQQ	TQ, left-edge quality, right-edge quality	TQ = min(255, round(10 × LLR)); it is not a posterior probability.
ddda_mcg.	Confident methylated-island interval	Whole-island molecule-specific annotation; not per-CpG methylation.
ddda_ucg.	Confident unmethylated-island interval	High-precision whitelist for CpG footprint evidence in CpG-aware SsDddA footprint recall.

See Online Methods, Molecular Annotation encoding and FIRE interoperability, and the cited MA specification.

bioRxiv preprint doi: https://doi.org/10.64898/2026.10.29.762797; this version posted October 31, 2026. The copyright holder for this preprint (which was not certified by peer review) is the author/funder, who has granted bioRxiv a license to display the preprint in perpetuity. It is made available under a CC-BY 4.0 International license.

Supplementary Note 1 | Browser display, navigation and analysis performance

Display and navigation compared with IGV

FiberBrowser, IGV desktop 2.19.2 and igv.js 3.8.0 were measured on the same local *NAPA* inputs at 1,000, 10,000 and 50,000 molecules. Each tool, depth, sampling setting and evidence setting was tested in five serial fresh-application trials, with rotating tool order. The final comparison contains 180 selected trials; 170 completed and 10 ended in an application failure. Completed operations before a failure remain in the results. Unreached operations have no timing estimate.

Application failures occurred in the following planned conditions. Later operations in the same trial were unreached; completed earlier operations remain in the source tables and five-repeat summaries (Supplementary Table 2).

Supplementary Table 2 | Application failures in the display benchmark

Tool / depth	Mode / evidence	Failed operation	Failure kind	Trials
igv.js / 50000	all / basic	cold pan_half	v8_out_of_memory	5
igv.js / 50000	all / rich	cold pan_half	v8_out_of_memory	5

Display measurement methods

The depth-controlled BAMs were constructed from 2,971 primary mapped *NAPA* DAF-seq templates spanning the locus center. Balanced replication assigned unique names, preserved alignment positions and optional tags, and introduced no positional jitter. The tested window was chr19:47514150–47516150 (zero-based, half-open). Input hashes and the construction script accompany the source tables. These synthetic-depth inputs measure scaling of this amplicon workload.

Each application opened the BAM and local reference at an empty locus before timing. The first sequence visited the target, panned by half its width, zoomed to half its width, then zoomed to twice its width about the original center. The same sequence was repeated after visiting the empty locus in the same process. Filesystem caches remained warm. Untimed pilots populated shared compiled-code caches. Application startup is recorded separately and excluded from the display comparison.

Sampling-on trials used a 4,000-read working target in FiberBrowser, explicit IGV sampling at 100 alignment starts per 50-base window, and the igv.js default sampling depth of 1,000. Their sampling algorithms produce different cohorts; actual loaded, displayed and drawn counts are retained. The configured FiberBrowser working target does not cap its direct-read path: in the dense rich trials, this path loaded the full region before selecting displayed reads. All-read trials disabled sampling, minimum-footprint filters and terminal-gap filters. Eligible primary, mapped, nonduplicate records were counted independently from the BAM.

FiberBrowser and igv.js ran in headless Chromium 147.0.7727.15 through Playwright 1.59.1. Browser viewports were 1500×1000 logical pixels at device scale 1 with one-pixel rows. The IGV desktop frame was 1500×1000 and used its stock adaptive squished layout. All-read initial and revisit captures verified every eligible name over bounded tiles (browser axes at most 8192 pixels; IGV capture height at most 4096). Navigation captures verified the visible

bioRxiv preprint doi: https://doi.org/10.64898/2026.10.29.762797; this version posted October 31, 2026. The copyright holder for this preprint (which was not certified by peer review) is the author/funder, who has granted bioRxiv a license to display the preprint in perpetuity. It is made available under a CC-BY 4.0 International license.

viewport. Stock draw calls, nonblank row pixels and PNGs provided paint evidence. Renderer implementations were unchanged.

The source tables separate target readiness, first visible PNG and completion of all-row capture with verification. Each process tree was sampled for memory every 100 ms. Peak values are summed resident-set sizes across tool and harness processes for the entire trial, including setup and both sequences. Shared pages can contribute to multiple processes; these values are not unique physical-memory allocations.

Trials requested a 16 GiB Java heap and a 16,384 MiB Chromium old-space limit. Effective heap limits are recorded separately because Chromium can impose a lower limit. Failures and subsequent unreached operations are tabulated without imputation. Medians and inclusive interquartile ranges are computed only for groups with all five repeats (Supplementary Table 3).

Supplementary Table 3 | All-read display times

Molecules	Tool / evidence	Visit	Median [IQR], s
1000	FiberBrowser / basic	cold	0.373 [0.370–0.376]
10000	FiberBrowser / basic	cold	3.253 [3.243–3.261]
50000	FiberBrowser / basic	cold	16.109 [15.733–16.259]
1000	IGV desktop / basic	cold	0.472 [0.467–0.478]
10000	IGV desktop / basic	cold	2.695 [2.693–2.724]
50000	IGV desktop / basic	cold	15.352 [15.186–15.355]
1000	igv.js / basic	cold	0.695 [0.688–0.696]
10000	igv.js / basic	cold	6.410 [6.398–6.430]
50000	igv.js / basic	cold	36.727 [36.635–36.789]
1000	FiberBrowser / basic	warm	0.153 [0.144–0.153]
10000	FiberBrowser / basic	warm	1.171 [1.170–1.172]
50000	FiberBrowser / basic	warm	5.679 [5.653–5.711]
1000	IGV desktop / basic	warm	0.323 [0.319–0.328]
10000	IGV desktop / basic	warm	2.280 [2.275–2.297]
50000	IGV desktop / basic	warm	14.536 [14.477–14.565]
1000	igv.js / basic	warm	0.658 [0.658–0.659]
10000	igv.js / basic	warm	6.230 [6.205–6.240]
50000	igv.js / basic	warm	Unreached / failed (0/5 completed)
1000	FiberBrowser / rich	cold	0.999 [0.995–1.008]
10000	FiberBrowser / rich	cold	7.457 [7.445–7.462]
50000	FiberBrowser / rich	cold	34.652 [34.605–34.776]
1000	IGV desktop / rich	cold	1.297 [1.296–1.302]
10000	IGV desktop / rich	cold	6.838 [6.760–6.852]
50000	IGV desktop / rich	cold	32.756 [32.722–32.896]
1000	igv.js / rich	cold	0.905 [0.902–0.912]

bioRxiv preprint doi: https://doi.org/10.64898/2026.10.29.762797; this version posted October 31, 2026. The copyright holder for this preprint (which was not certified by peer review) is the author/funder, who has granted bioRxiv a license to display the preprint in perpetuity. It is made available under a CC-BY 4.0 International license.

Molecules	Tool / evidence	Visit	Median [IQR], s
10000	igv.js / rich	cold	8.699 [8.686–8.712]
50000	igv.js / rich	cold	50.217 [50.111–50.231]
1000	FiberBrowser / rich	warm	0.507 [0.505–0.507]
10000	FiberBrowser / rich	warm	3.134 [3.079–3.136]
50000	FiberBrowser / rich	warm	13.352 [13.271–13.357]
1000	IGV desktop / rich	warm	1.073 [1.054–1.087]
10000	IGV desktop / rich	warm	6.293 [6.283–6.404]
50000	IGV desktop / rich	warm	32.011 [31.919–32.072]
1000	igv.js / rich	warm	0.857 [0.854–0.868]
10000	igv.js / rich	warm	8.436 [8.406–8.462]
50000	igv.js / rich	warm	Unreached / failed (0/5 completed)

Analyses at *NAPA*

Analysis timings used real, unreplicated *NAPA* Hia5 and SsDddA BAMs re-called with FiberHMM 3.0. The 340-base interval was chr19:47514980–47515320. Fixed NRF1 (47515206–47515222) and E-box (47515079–47515102) features were used for clustering, filtering and footprint co-occupancy. All analysis coordinates are zero-based and half-open. The timings characterize FiberBrowser analyses; these operations were not compared with IGV.

A fresh application was used for each assay, operation and repeat. Timing began after verified read display and two continuous seconds with no pending or new read/analysis requests; this setup wait was excluded from analysis latency. The display cap was 200 reads, row height was three pixels, and minimum-footprint, footprint-quality, spanning and terminal-gap filters were disabled. Each preview used the exact IDs verified on the canvas. Each full refinement retained the original full scope without an analysis or preparation cap. Default evidence requirements and feature-coverage rules determined eligibility.

API analyses were timed from browser request start through JSON decoding, excluding result-chart painting. Sort and clustering ran a preview followed by full refinement; their combined time is the per-trial sum of those two request durations, excluding harness bookkeeping between requests. The full stage is therefore measured after preview. The repeated sequence ran in the same process with caches retained. Scan used only the displayed cohort.

Sort used accessibility in descending order. Filter selected reads with open accessibility over the fixed motif features. Combinatorial clustering used accessibility features with a threshold of 0.5. K-means (k=5) used TF footprint features with a 90-base subnucleosomal threshold. Footprint co-occupancy used the saved *NAPA* rules: TF footprints 10–89 bases long overlapping the NRF1 and E-box intervals, with motif discovery, sequences and profiles disabled. Exact requests are retained (Supplementary Tables 4 and 5).

bioRxiv preprint doi: https://doi.org/10.64898/2026.10.29.762797; this version posted October 31, 2026. The copyright holder for this preprint (which was not certified by peer review) is the author/funder, who has granted bioRxiv a license to display the preprint in perpetuity. It is made available under a CC-BY 4.0 International license.

Supplementary Table 4 | FiberBrowser analysis times for Hia5

Analysis	Scope	First, median [IQR] s	Repeat, median [IQR] s
Scan peaks	Displayed; 109 reads	0.010 [0.010–0.011]	0.009 [0.009–0.009]
Sort accessibility	Displayed; 109 reads	0.106 [0.105–0.108]	0.011 [0.011–0.011]
Sort accessibility	Preview (109) then full (109 reads)	0.210 [0.208–0.210]	0.016 [0.016–0.016]
Filter accessibility	Full; 109 reads	0.216 [0.215–0.217]	0.007 [0.007–0.007]
Combinatorial cluster	Displayed; 105 reads	0.097 [0.094–0.099]	0.014 [0.012–0.014]
Combinatorial cluster	Preview (105) then full (105 reads)	0.202 [0.197–0.205]	0.022 [0.019–0.022]
K-means cluster (k=5)	Displayed; 105 reads	0.953 [0.934–0.963]	0.025 [0.024–0.026]
K-means cluster (k=5)	Preview (105) then full (105 reads)	1.108 [1.090–1.118]	0.039 [0.039–0.042]
Footprint co-occupancy	Full; 109 reads	0.552 [0.551–0.555]	0.010 [0.009–0.010]

Hia5: 109 eligible primary alignments overlap the benchmark window; 109 reads were painted. Full Sort and clustering membership were checked against endpoint-matched reference molecule sets and independent interval-coverage calculations.

Supplementary Table 5 | FiberBrowser analysis times for SsDddA

Analysis	Scope	First, median [IQR] s	Repeat, median [IQR] s
Scan peaks	Displayed; 190 reads	0.012 [0.011–0.012]	0.008 [0.008–0.009]
Sort accessibility	Displayed; 190 reads	0.107 [0.105–0.107]	0.016 [0.015–0.016]
Sort accessibility	Preview (190) then full (2,976 reads)	0.883 [0.881–0.887]	0.030 [0.030–0.030]
Filter accessibility	Full; 2,976 reads	1.341 [1.341–1.347]	0.023 [0.022–0.026]
Combinatorial cluster	Displayed; 190 reads	0.110 [0.110–0.115]	0.019 [0.019–0.021]
Combinatorial cluster	Preview (190) then full (2,966 reads)	0.939 [0.935–0.940]	0.083 [0.081–0.084]
K-means cluster (k=5)	Displayed; 190 reads	0.982 [0.970–0.988]	0.028 [0.028–0.030]
K-means cluster (k=5)	Preview (190) then full (2,965 reads)	2.030 [2.023–2.032]	0.092 [0.089–0.093]
Footprint co-occupancy	Full; 2,975 reads	1.430 [1.427–1.433]	0.026 [0.024–0.029]

SsDddA: 2,976 eligible primary alignments overlap the benchmark window; 190 reads were painted. Full Sort and clustering membership were checked against endpoint-matched reference molecule sets and independent interval-coverage calculations.

All analysis trials passed validation. First/repeat API payloads and complete scientific results were compared with timing fields excluded; five-repeat stability was checked separately for each operation. Working molecule sets were retrieved only after timed requests. Full Sort included every native eligible name, full clustering included all names covering both selected features, and co-occupancy retained the full molecule set. Network timelines were checked

bioRxiv preprint doi: https://doi.org/10.64898/2026.10.29.762797; this version posted October 31, 2026. The copyright holder for this preprint (which was not certified by peer review) is the author/funder, who has granted bioRxiv a license to display the preprint in perpetuity. It is made available under a CC-BY 4.0 International license.

for concurrent read or analysis requests. Raw responses and painted IDs accompany the source data.

Reproducibility

The Mac run used an Apple M3 Max, macOS-26.7.1-arm64-arm-64bit, 16 physical CPU cores and 64 GiB RAM, with Python 3.10.13. Software was FiberBrowser 3.0.0 and FiberHMM 3.0.0. Exact source commits, Chromium, Playwright, Java and library versions, source hashes and exact input hashes are retained with the run metadata. The packaged Mac application uses its bundled Python runtime; these benchmarks ran from source.

Source tables include display measurements and outcomes for every planned operation, analysis measurements and stage timings, cohort validation, input manifests, memory traces, screenshots, exact API payloads and failure logs. Pilots are retained and excluded from formal summaries.

bioRxiv preprint doi: https://doi.org/10.64898/2026.10.29.762797; this version posted October 31, 2026. The copyright holder for this preprint (which was not certified by peer review) is the author/funder, who has granted bioRxiv a license to display the preprint in perpetuity. It is made available under a CC-BY 4.0 International license.

Supplementary Note 2 | Complete-motif comparison of FiberHMM and ft footprint

FiberHMM footprints were compared with fibertools footprint calls (ft footprint) on the fixed panel of 512 CTCF motifs in each of K562, GM12878, H1, H9 and HepG2. The analysis used the literal 15-bp motif coordinates from the cohort catalog: [143,158) in plus-strand oriented windows and [142,157) in minus-strand windows. Both methods were scored on the same eligible molecule–motif observations, with library numerators and denominators pooled within each motif. ChIP labels were applied after calling. FiberHMM calls were scored by the same complete-motif rule as ft footprint.

FiberHMM positives required a footprint at LLR ≥5, width ≤100 bp, containing the complete motif (rule R1). Restricting widths to 10–89 bp (rule R3) gave exactly the same positive observations. Fibertools-rs v0.13.0 (commit 204f729) was run with one strand-oriented [0,15] module per motif, --ml 125 and -F 3844. A positive ft call was code 3, requiring an unmodified complete module within an original ft MSP. The common denominator contained 53,266, 41,529, 53,899, 49,553 and 26,694 eligible molecule–motif observations in K562, GM12878, H1, H9 and HepG2, respectively.

Site-level positive fractions were compared with cell-type-matched CTCF ChIP labels by AUROC. Confidence intervals are the 2.5th and 97.5th percentiles of 2,000 paired resamples of the 438 occupied 1-Mb genomic blocks. A block was defined by chromosome and floor(motif start/1,000,000); unique block labels were sorted lexicographically. NumPy default_rng (PCG64), seed 20260914, was reset per cell type, and the same saved draws were used for both callers. Paired difference intervals were calculated from the FiberHMM-minus-ft AUROC difference within each draw.

FiberHMM AUROCs ranged from 0.828 to 0.869 and ft footprint AUROCs from 0.810 to 0.875. All five paired 95% confidence intervals for the difference included zero (Supplementary Fig. 2e,f; Supplementary Table 6).

Supplementary Table 6 | CTCF complete-motif AUROCs and paired differences

Cell type	FiberHMM AUROC (95% CI)	ft AUROC (95% CI)	FiberHMM − ft (paired 95% CI)
K562	0.828 (0.791–0.865)	0.842 (0.804–0.877)	-0.014 (−0.036–0.007)
GM12878	0.829 (0.792–0.867)	0.810 (0.770–0.849)	0.019 (−0.005–0.045)
H1	0.869 (0.834–0.903)	0.875 (0.842–0.906)	-0.006 (−0.026–0.013)
H9	0.838 (0.802–0.873)	0.845 (0.811–0.880)	-0.007 (−0.030–0.014)
HepG2	0.855 (0.817–0.890)	0.867 (0.832–0.898)	-0.012 (−0.035–0.010)

Confidence intervals use paired genomic-block resampling as described in Supplementary Note 2. Values are rounded to three decimal places; paired differences were calculated before rounding.
